# Fundamental immune–oncogenicity trade-offs define driver mutation fitness

**DOI:** 10.1038/s41586-022-04696-z

**Published:** 2022-05-11

**Authors:** David Hoyos, Roberta Zappasodi, Isabell Schulze, Zachary Sethna, Kelvin César de Andrade, Dean F. Bajorin, Chaitanya Bandlamudi, Margaret K. Callahan, Samuel A. Funt, Sine R. Hadrup, Jeppe S. Holm, Jonathan E. Rosenberg, Sohrab P. Shah, Ignacio Vázquez-García, Britta Weigelt, Michelle Wu, Dmitriy Zamarin, Laura F. Campitelli, Edward J. Osborne, Mark Klinger, Harlan S. Robins, Payal P. Khincha, Sharon A. Savage, Vinod P. Balachandran, Jedd D. Wolchok, Matthew D. Hellmann, Taha Merghoub, Arnold J. Levine, Marta Łuksza, Benjamin D. Greenbaum

**Affiliations:** 1 Computational Oncology, Department of Epidemiology & Biostatistics, Memorial Sloan Kettering Cancer Center, New York, NY USA; 2 Swim Across America Laboratory and Ludwig Collaborative, Immunology Program, Parker Institute for Cancer Immunotherapy, Memorial Sloan Kettering Cancer Center, New York, NY USA; 3 Department of Medicine, Weill Cornell Medical College, New York, NY USA; 4 Parker Institute for Cancer Immunotherapy, Memorial Sloan Kettering Cancer Center, New York, NY USA; 5 Immunology and Microbial Pathogenesis Program, Weill Cornell Graduate School of Medical Sciences, New York, NY USA; 6 Hepatopancreatobiliary Service, Department of Surgery, Memorial Sloan Kettering Cancer Center, New York, NY USA; 7 Department of Medicine, Memorial Sloan Kettering Cancer Center, New York, NY USA; 8 Division of Cancer Epidemiology and Genetics, Clinical Genetics Branch, National Cancer Institute, National Institutes of Health, Rockville, MD USA; 9 Department of Pathology and Laboratory Medicine, Memorial Sloan Kettering Cancer Center, New York, NY USA; 10 Kravis Center for Molecular Oncology, Memorial Sloan Kettering Cancer Center, New York, NY USA; 11 Experimental and Translational Immunology, Health Technology, Technical University of Denmark, Lyngby, Denmark; 12 Physiology, Biophysics & Systems Biology, Weill Cornell Medicine, Weill Cornell Medical College, New York, NY USA; 13 Department of Surgery, Memorial Sloan Kettering Cancer Center, New York, NY USA; 14 Adaptive Biotechnologies, Seattle, WA USA; 15 David M. Rubenstein Center for Pancreatic Cancer Research, Memorial Sloan Kettering Cancer Center, New York, NY USA; 16 Human Oncology and Pathogenesis Program, Memorial Sloan Kettering Cancer Center, New York, NY USA; 17 Thoracic Oncology Service, Memorial Sloan Kettering Cancer Center, New York, NY USA; 18 Simons Center for Systems Biology, Institute for Advanced Study, Princeton, NJ USA; 19 Department of Oncological Sciences, Tisch Cancer Institute, Icahn School of Medicine at Mount Sinai, New York, NY USA

**Keywords:** Tumour immunology, Tumour-suppressor proteins, Computational models, Evolutionary theory

## Abstract

Missense driver mutations in cancer are concentrated in a few hotspots^1^. Various mechanisms have been proposed to explain this skew, including biased mutational processes^2^, phenotypic differences^3–6^ and immunoediting of neoantigens^7,8^; however, to our knowledge, no existing model weighs the relative contribution of these features to tumour evolution. We propose a unified theoretical ‘free fitness’ framework that parsimoniously integrates multimodal genomic, epigenetic, transcriptomic and proteomic data into a biophysical model of the rate-limiting processes underlying the fitness advantage conferred on cancer cells by driver gene mutations. Focusing on *TP53*, the most mutated gene in cancer^1^, we present an inference of mutant p53 concentration and demonstrate that *TP53* hotspot mutations optimally solve an evolutionary trade-off between oncogenic potential and neoantigen immunogenicity. Our model anticipates patient survival in The Cancer Genome Atlas and patients with lung cancer treated with immunotherapy as well as the age of tumour onset in germline carriers of *TP53* variants. The predicted differential immunogenicity between hotspot mutations was validated experimentally in patients with cancer and in a unique large dataset of healthy individuals. Our data indicate that immune selective pressure on *TP53* mutations has a smaller role in non-cancerous lesions than in tumours, suggesting that targeted immunotherapy may offer an early prophylactic opportunity for the former. Determining the relative contribution of immunogenicity and oncogenic function to the selective advantage of hotspot mutations thus has important implications for both precision immunotherapies and our understanding of tumour evolution.

## Abstract (web summary)

A mathematical framework to estimate the fitness of cancer driver mutations by integrating mutational bias, oncogenicity and immunogenicity finds fundamental trade-offs in cancer evolution.

## Main

The distribution of mutations in cancer is highly non-uniform. Mutations in oncogenes and tumour suppressors are enriched across cancers, and specific sites known as hotspots are more frequently mutated, leading to the hypothesis that hotspot mutations offer a selective advantage^1^. A paradigmatic example is the tumour suppressor p53. Although *TP53* is mutated in more than 50% of cancers, only eight hotspot mutations make up approximately one-third of all missense *TP53* mutations^3^. Several hypotheses have been offered to explain the mechanisms behind this skewed distribution, including biased generative mutational processes during tumour evolution^2,3^, degree of functional alteration^3–5^, structural stability^3,6^ and immune editing^7,8^. However, these hypotheses are not mutually exclusive. Mutations and subsequent selection can lead to substantial alterations in the concentration of oncogenic proteins^9–11^, a factor that has not been quantified as a contributor to the predominance of hotspot mutations. Generally, mutant p53 is present at a higher concentration than wild-type protein, depending on the tissue, copy-number alteration and mutation^12–14^. Yet, divergence from self and overexpression can contribute to mutant p53 neoantigen immunogenicity, constraining the ability of mutant p53 to avoid immune surveillance. Because neoantigens from mutations in tumour driver genes that are shared across patients and tumour types represent attractive immunotherapeutic targets^15,16^, understanding this issue is of critical importance. Here we examine the relationship between oncogenicity and immunogenicity for tumour driver mutations, using p53 as a primary example, to develop a model for predicting therapeutic targeting strategies, such as for neoantigen-based immune therapies.

**Fig. 1:**
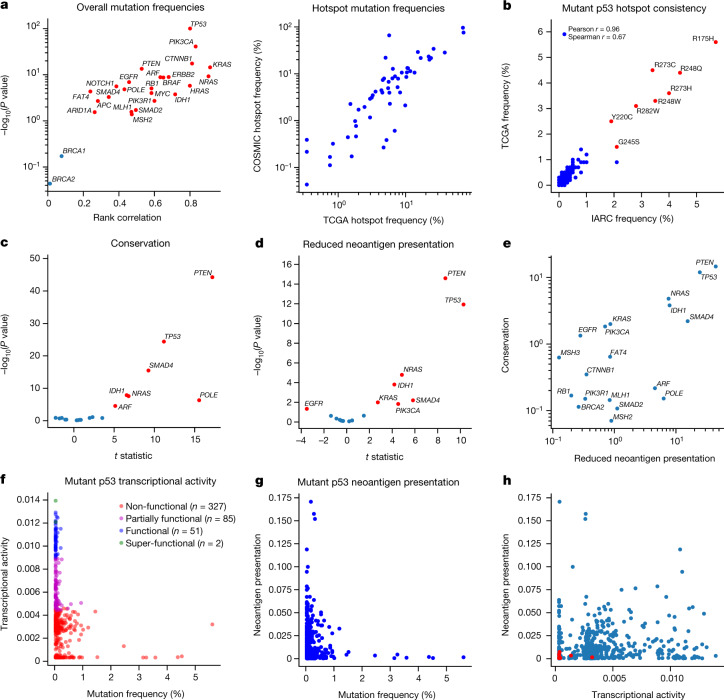
Driver gene hotspots are highly conserved and have relatively poor neoantigen presentation. **a**, Left, rank correlation between shared mutation frequencies in TCGA and the Catalogue of Somatic Mutations in Cancer (COSMIC) database for commonly mutated tumour suppressors and oncogenes plotted against the −log_10_-transformed rank correlation *P* value. Points corresponding to *P* < 0.05 are coloured red. Right, correlation of individual hotspot mutation frequencies in TCGA and the COSMIC database, excluding TCGA samples (Pearson *r* = 0.860, *P* < 0.0001; Spearman *r* = 0.851, *P* < 0.0001). **b**, Comparison of *TP53* mutation distributions in the TCGA (*n* = 2,764) and IARC (*n* = 21,170) databases (Pearson *r* = 0.963, *P* < 0.0001; Spearman *r* = 0.672, *P* < 0.0001; labelled hotspots coloured in red). **c**, Comparison of conservation in hotspots and other mutations in the same gene (Welch’s *t*-test *P* value, *P* < 0.05 annotated in red). **d**, Comparison of reduced neoantigen presentation between hotspots and other mutations in the same gene (Welch’s *t*-test *P* value, *P* < 0.05 annotated in red). **e**, −log_10_ *P* values from **c** and **d** plotted against each other. **f**, Mutant p53 transcriptional activity defined as the median of the inferred association constant for transcription factor affinity across eight transcriptional targets (*WAF1*, *MDM2*, *BAX*, *h1433s*, *AIP1*, *GADD45*, *NOXA* and *P53R2*) plotted against the frequency of *TP53* mutations in TCGA (Pearson *r* = −0.204, *P* < 0.0001; Spearman *r* = −0.404, *P* < 0.0001). **g**, Neoantigen presentation defined as effective mutant peptide affinity versus mutation frequency in TCGA (Pearson *r* = −0.079, *P* = 0.088; Spearman *r* = −0.053, *P* = 0.256; hotspots coloured in red). **h**, Mutant p53 transcriptional activity plotted against neoantigen presentation shows weak dependence between the two features (Pearson *r* = 0.073, *P* = 0.117; Spearman *r* = 0.144, *P* = 0.002; hotspots coloured in red).

**Extended Data Fig. 1:**
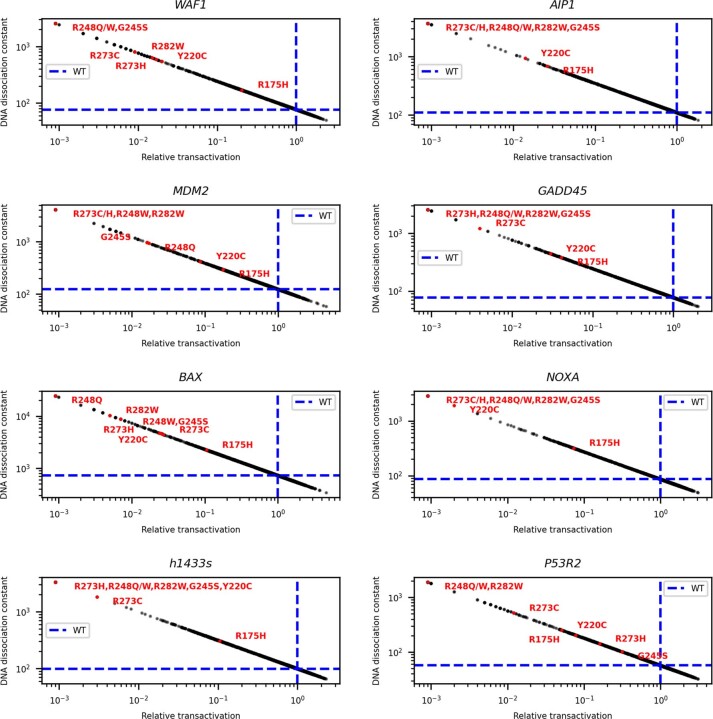
Inferred relationships between relative transactivation and apparent dimer dissociation constant. Relationship between the relative transactivation and the inferred apparent dimer dissociation constant for mutant homodimer p53. Blue dotted lines correspond to wild-type p53, which has a relative transactivation of 1 (Methods). The hotspots’ inferred values are annotated in red.

We found that mutation frequency distributions for commonly mutated driver genes were conserved across multiple cancer mutation databases (Fig. 1a, b) and that innate mutation rates based on trinucleotide context significantly correlated with mutation frequencies for several genes (Supplementary Information). We next quantified amino acid conservation over homologous proteins, a proxy for functional phenotype (Fig. 1c), and in silico*-*predicted reduced neoantigen presentation by major histocompatibility complex class I (MHC-I) molecules (Fig. 1d) across driver genes^7^. Several genes have hotspots at conserved sites and are poorly presented (Fig. 1e), implying that the fitness advantages the mutations confer may be driven by both features. We focused on *TP53* because it is widely mutated in tumours, with well-established, order-conserved pan-cancer hotspots (Fig. 1b and Supplementary Table 1) and broadly available functional phenotypic data^5^. We quantified the altered transcription factor function of mutant p53 across eight principal transcriptional targets with a quantitative yeast assay^5^ (Fig. 1f and Extended Data Fig. 1). We found that, although loss of transactivation was present for hotspot mutations, many non-hotspot mutations had comparatively low transactivation capacity. Moreover, we predicted MHC-I molecule presentation for the set of nonamer neopeptides surrounding p53 hotspot mutations to be worse than for non-hotspot peptides in The Cancer Genome Atlas (TCGA; *P* = 4.748 × 10^–7^, two-sided Welch’s *t*-test; Fig. 1g). Mutant p53 loss of transcriptional activity and neoantigen presentation of derived neopeptides showed only weak rank correlation (Fig. 1h), leading us to conclude that all of the mechanisms proposed to underlie mutant p53 fitness are likely to provide some predictive information.

**Fig. 2:**
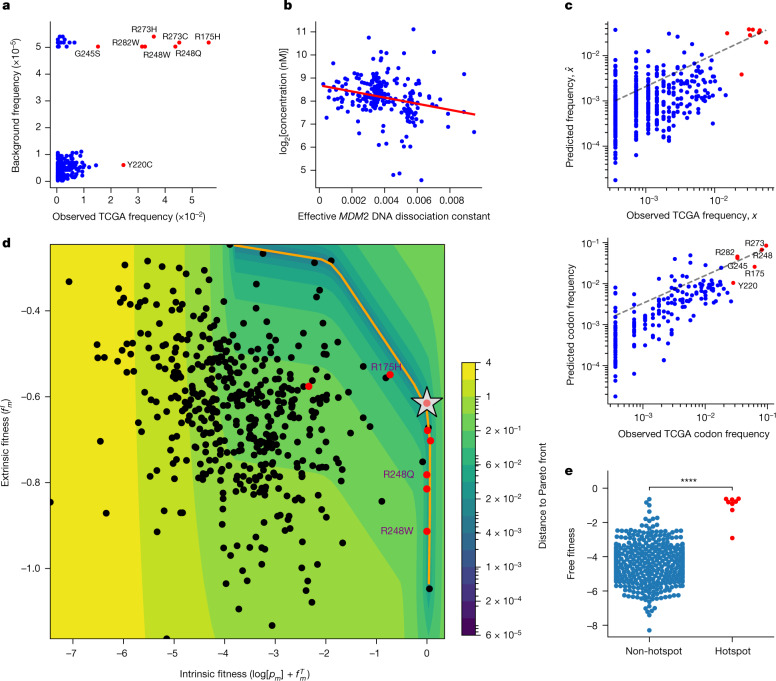
Mutant p53 fitness model quantifies the trade-off between oncogenicity and immunogenicity. **a**, Model with only background intrinsic mutational frequencies (Kullback–Leibler divergence, 1.222; Pearson *r* = 0.324, *P* < 0.0001; Spearman *r* = 0.2, *P* < 0.0001; hotspots coloured in red). **b**, Relationship between mutant p53 concentration (log_2_ transformed) and the predicted effective p53 association constant for the *MDM2* promoter across TCGA (*n* = 219; Pearson *r* = −0.25, *P* < 0.001; Spearman *r* = −0.29, *P* < 0.0001). **c**, Correlation of predicted *TP53* mutation frequencies to observed frequencies on a per-mutation basis (top; Kullback–Leibler divergence, 0.599; Pearson *r* = 0.671, *P* < 0.0001; Spearman *r* = 0.39, *P* < 0.0001) and per-protein position basis (bottom; Kullback–Leibler divergence, 0.337; Pearson *r* = 0.794, *P* < 0.0001; Spearman *r* = 0.782, *P* < 0.0001). **d**, Sum of the log-transformed background frequency log[*p*_m_] and positive functional fitness ${f}_{m}^{T}$, denoted intrinsic fitness, plotted against negative immune fitness (${f}_{m}^{I}$, extrinsic fitness) (Pearson *r* = −0.31, *P* < 0.0001; Spearman *r* = −0.33, *P* < 0.0001). The orange line corresponds to the Pareto front; the silver star indicates optimal free fitness constrained by the Pareto front; and the heat map corresponds to the distance to the Pareto front. The hotspot mutations are coloured red and the R175H and R248Q/W mutations are shown. **e**, Comparison of the free fitness distributions of non-hotspot and hotspot mutations (*P* < 0.0001, Welch’s *t*-test).

**Extended Data Fig. 2:**
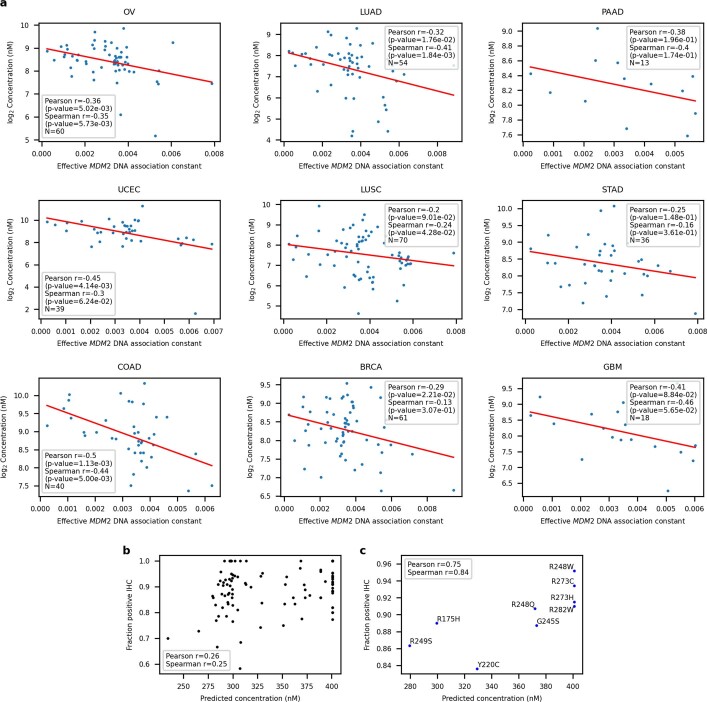
Relationship between mutant p53 concentration and predicted *MDM2* binding affinities. **a**, Variation in normalized concentration across mutant p53 versus predicted affinity to *MDM2* DNA in common *TP53*-mutated tissues within TCGA. Protein concentration is expressed as log2 of inferred protein concentration in nanomolar (nM) units. **b**, Fraction positive immunohistochemistry (IHC) assay from the IARC R20 dataset plotted against predicted per-allele mutant p53 concentration averaged across tissues. Correlations are for mutations with at least 10 IHC data entries (Pearson p-value 0.00848, Spearman p-value 0.00967). **c**, Fraction positive IHC assay plotted against predicted per-allele mutant p53 concentration averaged across tissues only for mutant *TP53* hotspots (Pearson p-value 0.0207, Spearman p-value 0.00503).

**Extended Data Fig. 3:**
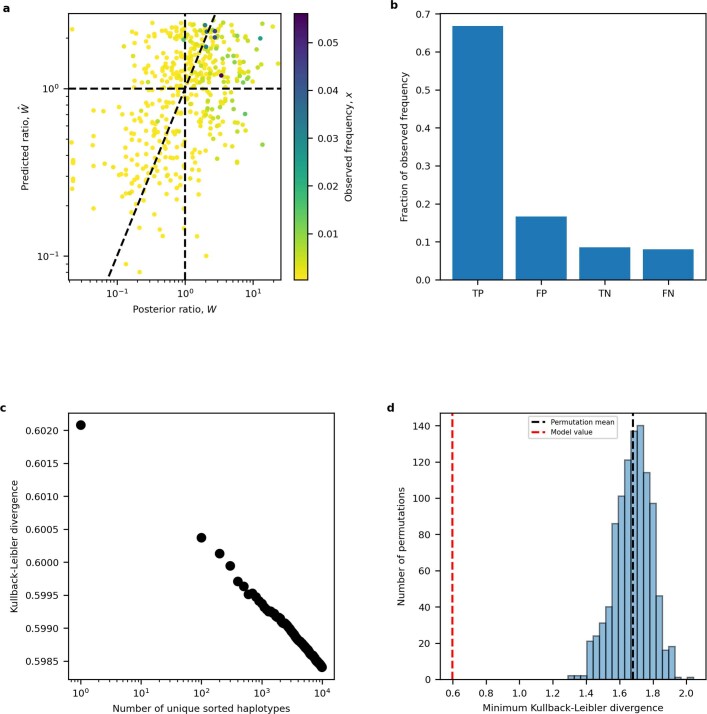
Fitness model prediction analysis. **a**, Predicted ratio from combined fitness model plotted against posterior ratio for each *TP53* mutation. Mutations are colored by their observed frequency. Ratios > 1 are predicted to be fixed in the cancer population. Diagonal line corresponds to ratios being equal. **b**, Prediction accuracy plotted as the proportion of observed mutation frequency for true positive (TP), false positive (FP), true negative (TN) and false negative (FN) model predictions. **c**, Kullback-Leiber divergence versus number of simulated HLA-I haplotypes shows improved model predictions according to the haplotype sample size. **d**, Internal validation by shuffling background mutation frequencies, functional phenotypes and immune phenotypes of *TP53* mutations for 1,000 iterations and computing the Kullback-Leibler divergence for each iteration. The histogram is of the distribution of Kullback-Leibler divergences from all iterations. Permutation-mean Kullback-Leibler divergence is plotted as a vertical black dotted line and the true Kullback-Leibler divergence is plotted as a vertical red dotted line.

**Extended Data Fig. 4:**
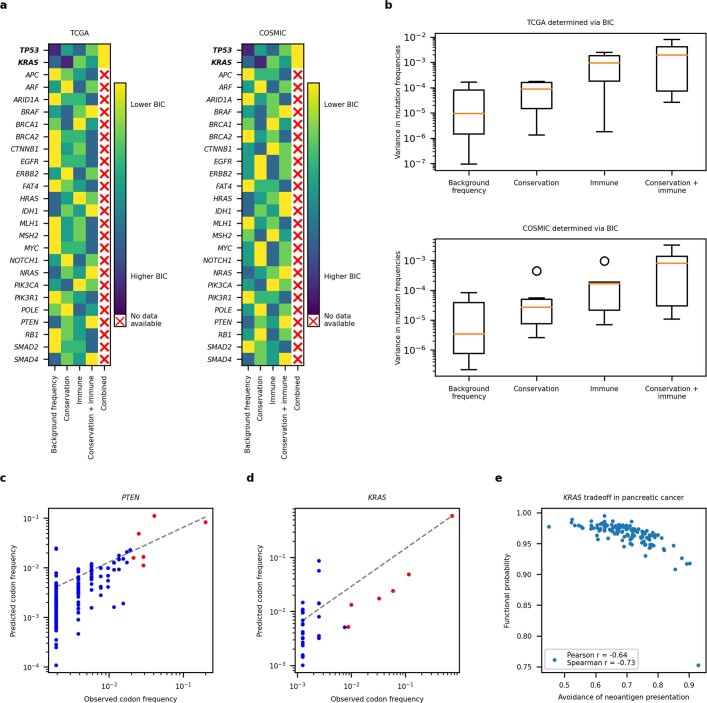
Fitness model predicts mutation frequencies in commonly mutated cancer driver genes. **a**, Degree to which models of varying complexity account for mutation distributions from TCGA and COSMIC, excluding TCGA samples, across 27 commonly mutated cancer driver genes. Models are ranked by Bayesian Information Criterion (BIC) in descending order (models with the lowest BIC value are deemed the most explanatory). **b**, Boxplots of observed mutation frequency variances of driver genes best explained by a particular model, ranked by complexity in ascending order. **c**, Fitness model results for *PTEN* per protein position in TCGA, using both conservation and immunogenicity over background mutation rates. The full model is justified by the BIC value (KL divergence = 0.269; Pearson r = 0.701, p-value = 2.013e-24; Spearman r = 0.701, p-value = 2.386e-24). **d**, Fitness model results for *KRAS* per protein position in TCGA, using a full model with conservation, function and immunogenicity over background mutation rates with functional information available for seven frequent *KRAS* cancer mutations (G12A/C/D/R/V, G13D and Q61L). All components are justified by the BIC value (KL divergence = 0.256; Pearson r = 0.981, p-value = 2.095e-24; Spearman r = 0.616, p-value = 0.000104). **e**, Trade-off between gain-of-function and avoidance of neoantigen presentation, defined as $1-{I}_{m}\left(H\right)$, in TCGA pancreatic cancer for *KRAS* hotspots (Pearson −0.750, p-value = 2.599e-23; Spearman r = −0.774, p-value = 1.507e-25). Each point corresponds to an individual pancreatic cancer sample with a hotspot *KRAS* mutation.

We therefore sought to harmonize this proposed feature set within a mechanistic mathematical model of mutant p53 fitness^17–21^. A model based on background mutation rates alone was insufficient to separate the hotspots from other mutations (Fig. 2a). We further looked to capture variation in mutant p53 concentration, which affects both the transcription factor function and neoantigen presentation. We assigned TCGA samples a normalized p53 protein concentration and effective *MDM2* promoter affinity to infer typical per-allele mutant-specific concentrations^22,23^. We consistently found a significant inverse relationship between these two variables across tumour types (Fig. 2b and Extended Data Fig. 2a) and a significant correlation between our concentration estimates and immunohistochemistry data (Extended Data Fig. 2b, c). We constructed a nonlinear, two-parameter model that separates mutant p53 fitness onto a positive pro-oncogenic probability and a negative immunogenic probability (Supplementary Methods) coupled to mutant p53 concentration. Each component is given an appropriate weight by maximum-likelihood fitting with respect to TCGA mutation frequencies. Our fitness model successfully predicts the distribution of mutation frequencies, both per mutation and per codon (Fig. 2c and Supplementary Information), and accurately predicts the increase or decrease in each mutant frequency with respect to background frequency (Extended Data Fig. 3a, b). We found that predicting the distribution of *TP53* mutations requires both functional and immune components through determining the relative likelihoods of the models (Supplementary Table 2 and Supplementary Methods). Model optimization depended strongly on the sampled MHC-I haplotype and all mutant phenotypes (Extended Data Fig. 3c, d and Supplementary Information). We optimized and applied similar models to other driver genes, with conservation used as a proxy for function (Extended Data Fig. 4a and Supplementary Methods). Combined models were more predictive for mutation distributions with larger frequency variance across all database mutations, which implies that increased mutation frequency variance relates to increased selection, as expected from Fisher’s theorem^24^ (Extended Data Fig. 4b), such as for *PTEN* (Extended Data Fig. 4c). To build a predictive model for *KRAS*, we were able to include measured binding affinities to the downstream Raf effector protein for a limited set of hotspot mutations^25^ (Supplementary Methods), in addition to inferences in conservation and immunogenicity (Extended Data Fig. 4d).

To represent the landscape of mutant p53 fitness, we defined a ‘free fitness’ function of each mutation as the sum of the positive functional fitness, the negative immune fitness and the logarithm of the background frequency (Supplementary Methods), analogous to a free energy in statistical physics with the multiplicity of states derived from the background mutation rate. We plotted the free fitness landscape (Fig. 2d) and observed a general trade-off between intrinsic fitness (logarithm of the background frequency and functional fitness; Supplementary Methods) and extrinsic immune fitness. The trade-off observed in *TP53* is reminiscent of other evolutionary trade-offs, and we theorized that *TP53* hotspots were Pareto optimal^26,27^. We computed the Pareto front and identified the optimal fitness coordinate constrained by the front when using our model (Fig. 2d and Supplementary Methods). We found that hotspots had statistically higher free fitness (Fig. 2e) and occupied an optimal regime in which they successfully trade off between the pro-tumorigenic benefit of functional loss and the cost of presenting immunogenic neoantigens. However, there was substantial variation among the hotspot mutations. For instance, R175H is functionally the most wild-type-like hotspot but typically has the poorest MHC-I binding capacity. By contrast, the R248Q and R248W (R248Q/W) mutations have nearly complete loss of transcriptional function and therefore can more often afford to generate potentially immunogenic neoantigens, because the proliferative competitive advantage induced by mutation would offset the cost of immunogenicity. For *KRAS*, under more restrictive assumptions, we observed evidence for a trade-off between functional and immune fitness for hotspot mutations in pancreatic adenocarcinoma, where *KRAS* is typically mutated (Extended Data Fig. 4e and Supplementary Methods).

**Extended Data Fig. 5:**
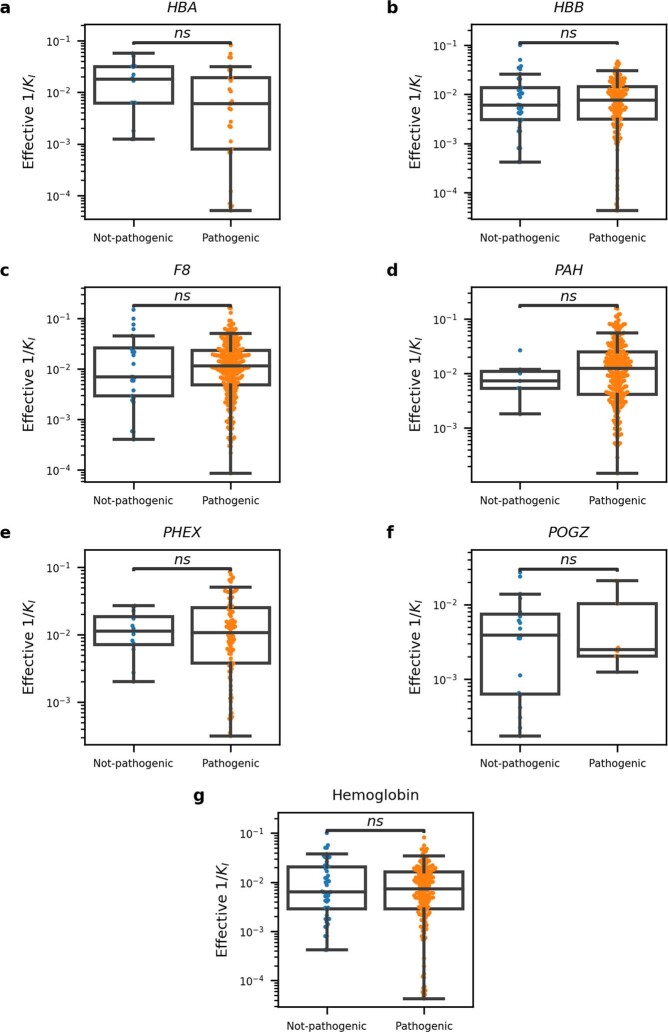
Inferred mutant immunogenicity is not related to pathogenicity in non-cancer driver genes. **a–f**, Comparison of inferred immunogenicity across not-pathogenic and pathogenic missense mutations in nine non-cancerous disease driver genes (*HBA*, *HBB*, *HBD*, *HG1*, *HG2*, *F8*, *PAH*, *PHEX* and *POGZ*) using the Mann-Whitney U-test. Six out of nine genes had sufficient data for comparison between not-pathogenic and pathogenic mutations (*HBA*, *HBB*, *F8*, *PAH*, *PHEX* and *POGZ*). **g**, Data corresponding to all hemoglobin subunits (*HBA*, *HBB*, *HBD*, *HG1* and *HG2*) were combined and compared (Hemoglobin). Mutations and their “Not-pathogenic” and “Pathogenic” status were determined using the NCBI’s dbSNP and ClinVar systems, respectively.

**Extended Data Fig. 6:**
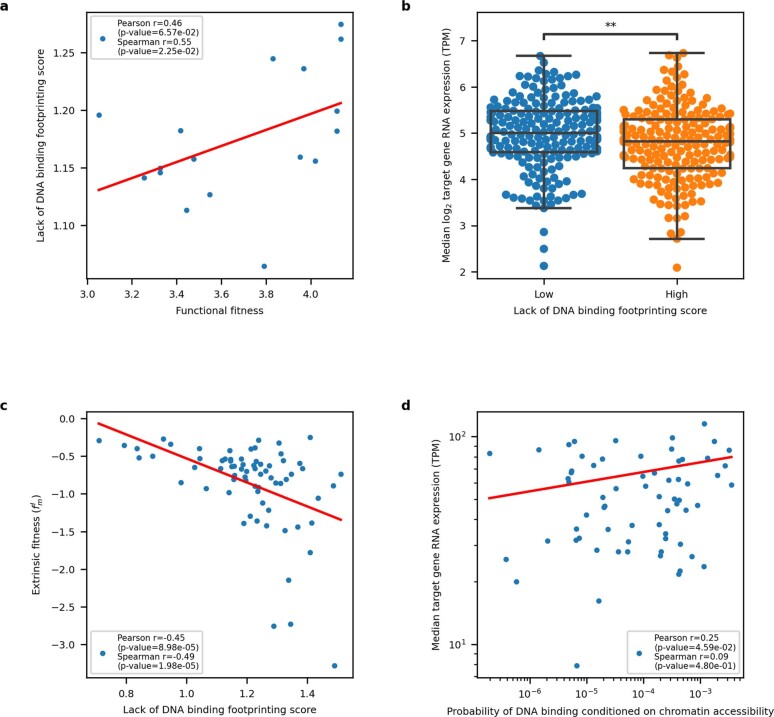
Fitness trade-offs inferred from ATAC- and RNA-seq. **a**, Lack of binding score plotted versus predicted functional fitness. Most TCGA ATAC-seq samples were breast cancers (BRCA), therefore we only plot matched BRCA samples to normalize on tissue-specific protein abundance (Pearson r = 0.46, p-value = 0.063, Spearman r = 0.55, p-value 0.023, N = 17). **b**, log_2_ of median TCGA RNA expression (TPM) of eight p53 target genes utilized in fitness model split on median TCGA ATAC-seq lack of DNA binding score (Mann-Whitney p-value = 0.006). **c**, Immune fitness plotted versus ATAC-seq-based lack of DNA binding footprinting score for each TCGA sample (Pearson r = −0.45, p-value < 0.0001; Spearman r = −0.49, p-value < 0.0001). **d**, Median TCGA RNA expression (TPM) of the target genes with available ATAC-seq data (*WAF1*, *BAX*, *h1433s*, *AIP1*, *GADD45* and *NOXA*) plotted versus median probability of mutant p53 binding DNA, conditioned on target DNA chromatin accessibility (Pearson r = 0.25, p-value 0.0459; Spearman r = 0.088, p-value 0.480).

One possible explanation for the inverse relationship is that mutations that alter protein function are generally more likely to generate differentially immunogenic peptides. We therefore compared non-pathogenic and pathogenic mutations in a curated set of non-cancerous disease driver genes and found that both types of mutation generated comparably predicted immunogenic peptides (Extended Data Fig. 5), implying that the trade-off observed is not to be expected a priori. Moreover, because our functional predictions for mutant *TP53* are based on precision yeast assays, we checked for evidence of an oncogenic–immunogenic trade-off using independent TCGA assay for transposase-accessible chromatin with sequencing (ATAC-seq) and RNA sequencing assay to develop a score for the lack of mutant p53 binding site occupancy (Supplementary Methods). We found that the functional component of our fitness model correlated significantly with lack of binding (Extended Data Fig. 6a) and that samples with increased lack of p53 binding consistently showed decreases in p53 target gene RNA expression (Extended Data Fig. 6b). We independently re-derived the oncogenicity–immunogenicity trade-off by comparing the inferred immunogenicity to our scores for lack of binding (Extended Data Fig. 6c). Finally, as a further control, we found a correlation between the yeast assay-derived probability of DNA binding and median target gene RNA expression conditioned on chromatin accessibility (Extended Data Fig. 6d).

**Fig. 3:**
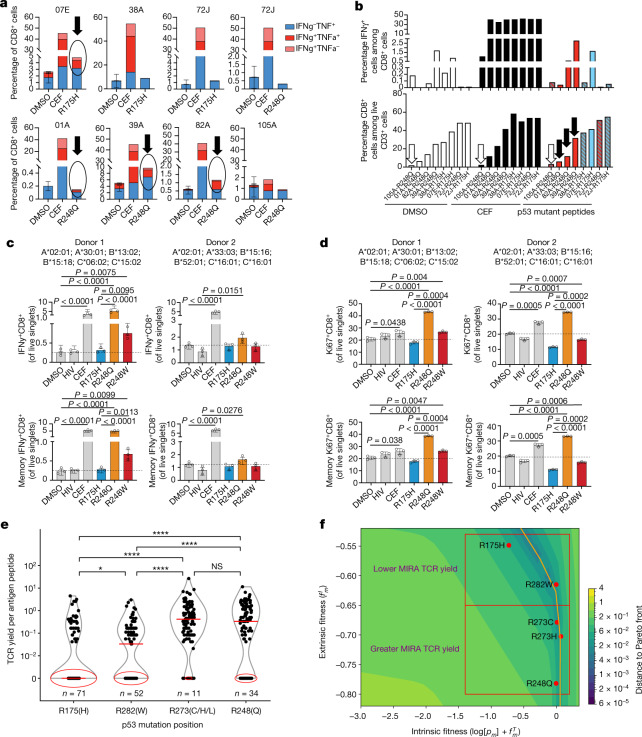
Validation of differential reactivity to mutant p53 neoepitopes in healthy donors and patients with cancer. **a**, **b**, PBMCs from patients with R175H and/or R248Q p53-mutant tumours were cultured with the indicated p53 neopeptides or with CEF or DMSO as positive and negative controls, respectively. **a**, Flow cytometry quantification of cells expressing IFNγ ± TNFα among CD8^+^CD3^+^ live T cells in the indicated samples. DMSO data are the mean ± s.d. of two to three technical replicates. **b**, Assessment of IFNγ responses (IFNγ^+^ cells among CD8^+^ T cells) in the same samples as in **a** in association with the frequencies of total CD8^+^ T cells in those cultures. Black arrows indicate reacting samples; a white arrow indicates low-input CD8^+^ T cells. **c–f**, Reactivity of PBMCs from healthy donors to the indicated p53 neoantigens by an optimized ex vivo priming assay (**c**, **d**) and MIRA assay using TCR sequencing to quantify specific T cell clonal expansion (**e–f**). IFNγ (**c**) and Ki67 (**d**) expression was assessed in the total CD8^+^ T cell fraction (top) or the non-naive memory CD8^+^ T cell fraction (bottom). Frequencies are shown for two individual healthy donors as the percentage of live single cells in culture after 2 weeks of in vitro stimulation with the indicated p53 neopeptides compared with CEF and DMSO or an HIV peptide pool as positive and negative controls, respectively. **e**, Quantification of reactive TCRs in 107 healthy donors in 222 MIRA assay experiments, with an average of two experiments per donor. Median values are denoted by red horizontal line; zero values are circled in red with the number of zero values annotated in blue. **f**, *TP53* hotspots tested in **e** along the Pareto front yielding fewer or more TCRs grouped in red squares. Statistical significance was assessed by unpaired two-sided *t*-tests (**c**, **d**) or Mann–Whitney *U*-test (**e**). *P ≤ 0.05, **P ≤ 0.01, ***P ≤ 0.001, ****P ≤ 0.0001.

**Extended Data Fig. 7:**
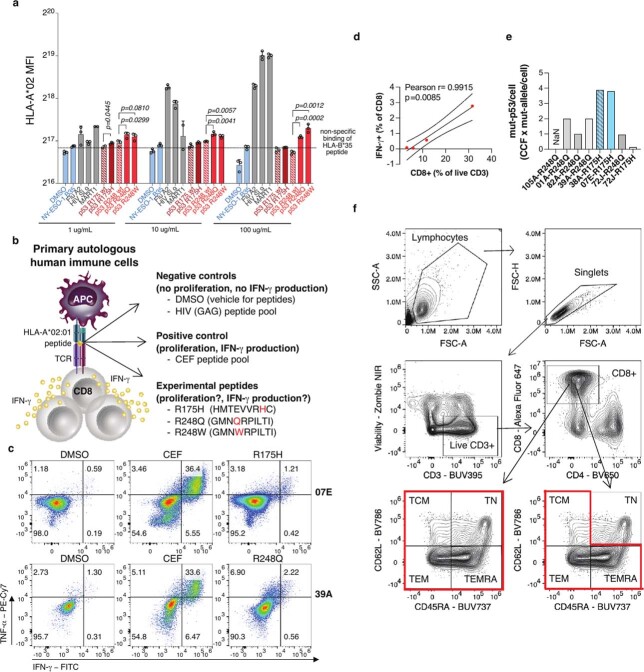
Differential T-cell reactivity to p53 neopeptides. **a**, Flow cytometry quantification of HLA-A*02:01 expression on the surface of live T2 cells as a measure of peptide:MHC stabilization via binding to specific peptides. T2 cells were incubated overnight in serum-free media with recombinant human B2M and the indicated peptides at the indicated concentrations, or DMSO as vehicle control. Blue, negative controls (DMSO and unrelated HLA-B*35-restricted NY-ESO-1-derived peptide); red, positive controls (HLA-A*02:01-restricted peptides from flu and HIV viral antigens and Mart1/Melan-A melanoma-associated antigen); gray, experimental peptides containing the indicated mutation in comparison with the corresponding wild-type (wt) sequence. Data are mean ± SD of 2-3 replicates. *P* values are calculated with a two-sided unpaired t-test. **b**, Model illustrating the molecular basis of the T-cell stimulation assay and stimulation conditions (APC, antigen presenting cell; TCR, T-cell receptor). **c**, Representative plots of IFN-γ ± TNF-a expressing cells among CD8+CD3+ live T cells in PBMCs from patients with mutant p53 tumors as in Fig. 3a. **d**, Correlation analyses between indicated parameters in PBMC samples from R248Q mutant patients with presence of disease (N = 4) at the time of PBMC collection as in Fig. 3b. **e**, Estimate of mutant p53 amount per tumor cell before treatment in the same patients. Samples with R175H mutations are colored in blue. The sample which reacted, corresponding to the patient who received immune checkpoint blockade (ICB) therapy, is in solid blue, and the sample which did not react, and did not receive ICB, has filled-in lines. **f**, Flow cytometry gating strategy for total CD8 and non-naïve memory CD8 T-cells analyzed in Fig. 3c, d. TN: naïve T-cells, TCM: central memory T-cells, TEM, effector memory T-cells, TEMRA: effector memory T-cells re-expressing CD45RA.

We tested our immunogenicity predictions for mutant p53 using peptides from hotspot mutations predicted to be presented on human leukocyte antigen (HLA)-A*02:01 (Supplementary Table 3 and Supplementary Methods), which is the most frequent MHC-I allele in TCGA. First, we asked whether these peptides had differential ability to bind and stabilize HLA on the cell surface, using the TAP2-deficient human lymphoblastoid T2 cell line (Supplementary Methods). We found that R248Q/W peptides but not R175H peptide could significantly stabilize HLA-A*02:01 expression on T2 cells in a dose-dependent manner in comparison with the respective wild-type peptide sequence (Extended Data Fig. 7a and Supplementary Table 3). We next asked whether R175H and R248Q/W *TP53* hotspot mutations elicit differential immune responses in vivo in patients with cancer. We identified seven HLA-A*02:01-positive patients with either bladder or ovarian tumours with these mutations and available peripheral blood mononuclear cell (PBMC) samples at Memorial Sloan Kettering Cancer Center (MSKCC). In total, three samples were from patients with R175H-mutant tumours (07E, 38A and 72J) and five samples were from patients with R248Q-mutant tumours (72J, 01A, 39A, 82A and 105A) (Supplementary Table 4). One patient’s tumour (72J) had both mutations, although the R175H clonal fraction was far lower (Supplementary Table 4). All but two patients (72J and 07E) were immunotherapy naive at the time of sample collection. Patient 72J, who had a tumour with both hotspot mutations, had an ongoing complete response to nivolumab (anti-programmed death (PD)-1) treatment with no disease detectable at the time of PBMC collection. Patient 07E, who harboured the R175H mutation, was on atezolizumab (anti-PD-L1) treatment at the time of PBMC collection. All other samples were collected before treatment initiation. We stimulated the PBMCs with peptides harbouring the R175H or R248Q mutations or with a CEF (cytomegalovirus, Epstein–Barr virus, and influenza virus) peptide pool or DMSO as positive and negative controls, respectively (Supplementary Table 3). We then measured the interferon-γ (IFNγ) and tumour necrosis factor-α (TNFα) production in CD8^+^ T cells by flow cytometry (Fig. 3a, b and Extended Data Fig. 7b). We found responses in three of the five R248Q samples, with the response proportional to the size of the CD8^+^ T cell population (Fig. 3a, b and Extended Data Fig. 7c, d). This indicates responses might correlate with the frequency of CD8^+^ T cell precursors recognizing the neopeptides. By contrast, only one of the three patients with R175H-mutant tumours had neopeptide reactivity; this patient (07E) had one of the largest expansions for the mutant *TP53* allele and a concomitant increase in protein abundance as well as a positive response to anti-PD-L1 treatment (Fig. 3a and Extended Data Fig. 7e). This finding in combination with the lack of T cell reactivity in the immunotherapy-naive patient (38A) with four mutant R175H alleles indicates despite expansion of the mutant allele, R175H tends to be less immunogenic than R248Q/W, but anti-R175H T cell responses may be unleashed by immune checkpoint blockade therapy. Consistent with this, we found no reactivity in patient 72J, who harboured both hotspot mutations at lower abundance (Extended Data Fig. 7e) and had a complete response to immune checkpoint blockade therapy. This indicates that, in cancer, expansion and/or persistence of cognate T cell pools depends on the levels of the mutant protein.

We next asked whether differential immunogenicity of *TP53* hotspots was a broad phenomenon in the healthy population and therefore potentially linked to the frequency of T cell precursors recognizing a mutant peptide. We compared the capacity of R175H and R248Q/W peptides when loaded onto autologous antigen-presenting cells to prime and expand specific T cells in two healthy donors with the HLA-A*02:01 allele (Extended Data Fig. 7b, Supplementary Table 3 and Supplementary Methods). We consistently noted greater IFNγ and Ki67 expression in T cells stimulated with R248Q/W peptides than in those stimulated with R175H peptides in both donors (Fig. 3c, d and Extended Data Fig. 7f). Furthermore, we assessed the yield of *TP53* hotspot-specific T cell clones by multiplex identification of T cell receptor (TCR) antigen specificity (MIRA) assay (Adaptive Biotechnologies) in PBMC samples from 107 healthy donors representing a set of distinct HLA alleles, including 25 *HLA-A*, 46 *HLA-B* and 20 *HLA-C* alleles (Supplementary Methods). Forty mutant epitopes from R175, R282, R273 and R248 loci covering the top six p53 hotspots were screened for multiple peptide lengths. The distribution of normalized TCR yield per antigen peptide per donor, indicative of specific clonal expansion, was plotted for each hotspot position (Fig. 3e). Notably, we found that the R175 hotspot yielded statistically lower TCR reactivity per peptide as compared with all other hotspots, having a median value of zero reacting TCRs per peptide. Moreover, we found that hotspot reactivity corresponded to fitness model predictions (Fig. 3f). These results indicate that the MHC-I haplotype and TCR repertoire distributions of the healthy population may be more likely to react to the R248 locus than the R175 locus.

**Fig. 4:**
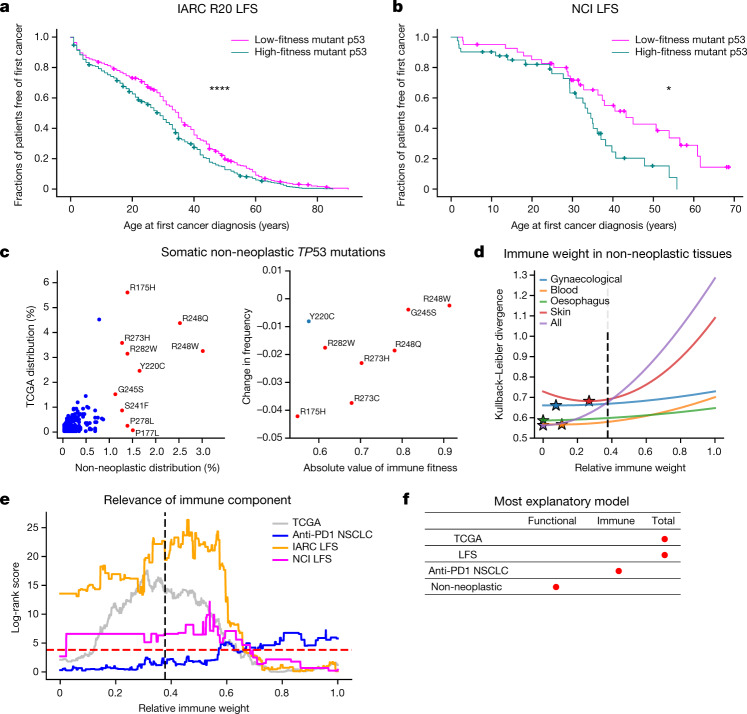
Mutant p53 fitness informs LFS age of tumour onset and non-neoplastic *TP53* mutation distribution. **a**, **b**, Kaplan–Meier curves split on median mutant p53 fitness from the combined model for age of tumour onset in the IARC R20 germline dataset (*n* = 998) (**a**) and the NCI LFS dataset (*n* = 82) (**b**). **c**, Left, comparison of *TP53* mutation frequencies in non-neoplastic tissues (3,451 mutation occurrences) and the frequencies in TCGA (2,764 mutation occurrences; Pearson *r* = 0.732, *P* < 0.0001; Spearman *r* = 0.544, *P* < 0.0001; top 10 non-neoplastic mutations coloured in red and annotated). Right, positive relationship between hotspot frequency difference in non-cancerous and cancerous cells and magnitude of immune fitness. CpG-associated hotspots are coloured in red; Y220C is coloured in blue (overall: Pearson *r* = 0.594, *P* = 0.120; Spearman *r* = 0.619, *P* = 0.102; CpG-associated hotspots only: Pearson *r* = 0.827, *P* = 0.022; Spearman *r* = 0.786, *P* = 0.036). **d**, Kullback–Leibler divergence plotted as a function of relative immune weight for the largest tissue-specific mutation distributions across collected non-neoplastic somatic p53 mutations. Optimal immune weights are denoted as stars, and the optimal relative immune weight derived independently to best represent the observed mutation frequency in TCGA is denoted as a black dotted line. **e**, Log-rank scores of the TCGA (*n* = 1,941), NSCLC (*n* = 289) and LFS (IARC, *n* = 946; NCI, *n* = 82) cohorts as a function of the relative immune weight. The dashed red line corresponds to the log-rank score for *P* = 0.05; the dashed black line marks the choice of parameters trained independently to best represent the observed mutation frequency in TCGA. **f**, The most explanatory models across mutant *TP53* datasets, as indicated by red dots.

**Extended Data Fig. 8:**
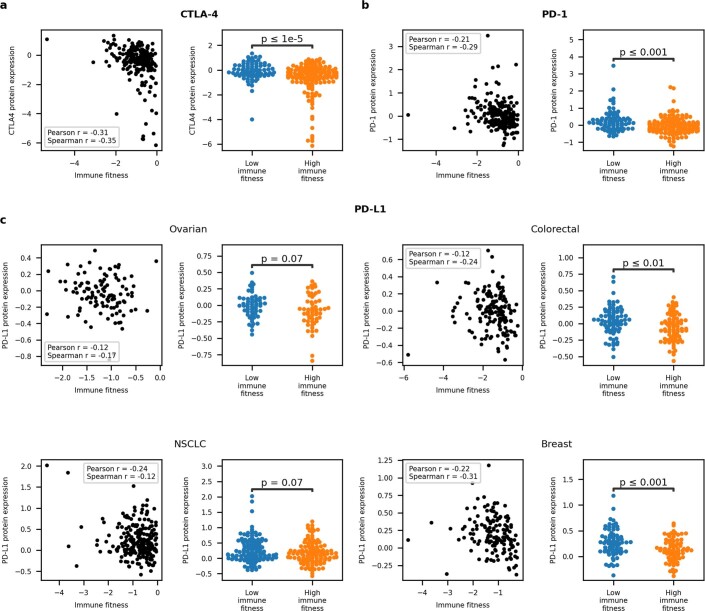
Relationships between immune fitness and immune checkpoint protein expression in TCGA. **a, b**, Continuous and categorical relationships between CTLA-4 **(a)** and PD-1 **(b)** protein expression available from TCGA RPPA proteomics assay and immune fitness. For the CTLA-4 scatterplot, Pearson p-value < 0.0001, Spearman p-value < 0.0001. For the PD-1 scatterplot, Pearson p-value = 0.00153, Spearman p-value < 0.0001. Categorical differences measured with the Welch’s t-test. **c**, Continuous and categorical relationships between PD-L1 protein expression available from TCGA RPPA proteomics assay and immune fitness in commonly *TP53*-mutated tissues. Correlation p-values: Ovarian - Pearson p-value = 0.2, Spearman p-value = 0.0829; Colorectal - Pearson p-value = 0.157, Spearman p-value 0.003; NSCLC - Pearson p-value = 0.0812, Spearman p-value = 0.00793; Breast - Pearson p-value = 0.00671, Spearman p-value = 0.000140. Categorical differences measured with the Welch’s t-test.

**Extended Data Fig. 9:**
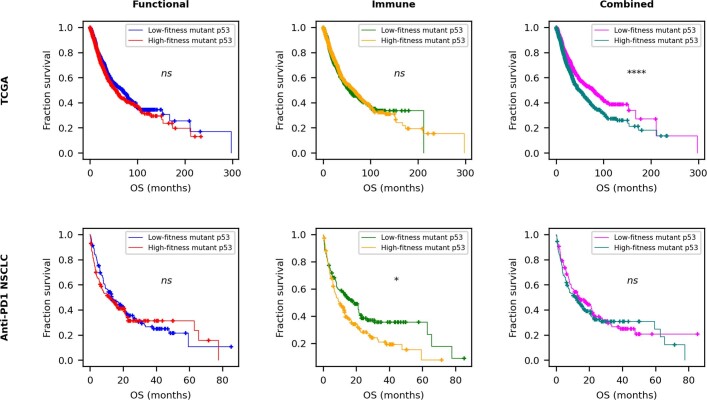
p53 fitness predicts survival and immune relevance in diverse p53-mutated groups. Kaplan-Meier curves separated by median functional, immune and total fitness in TCGA and MSKCC non-small cell lung cancer (NSCLC) ICB-treated samples. For NSCLC samples, matched HLA-*TP53* mutation pairs with lung-specific and allele-specific concentrations were used to determine functional, immune and combined fitness. *ns* p > 0.05, * p ≤ 0.05, ** p ≤ 0.01, *** p ≤ 0.001, **** p ≤ 0.0001.

Validating the link between increased immunogenicity and immune response to mutant p53, we found that the protein abundance of the CTLA-4, PD-1 and PD-L1 immune checkpoint proteins was higher in TCGA samples with *TP53* mutations that were predicted to be more immunogenic (Extended Data Fig. 8). Our results suggest increased immune activation and concurrent establishment of adaptive immune resistance. When we segregated survival on the basis of functional, immune and combined fitness in TCGA and a cohort of patients with non-small-cell lung cancer (NSCLC) treated with anti-PD-1 at MSKCC (Extended Data Fig. 9), we found that functional and immune fitness components were required to achieve significant survival separation in TCGA, whereas immune fitness on its own significantly separated immunotherapy-treated patients with NSCLC by survival. For robustness, we retrained our models across a range of relative weights between functional and immune fitness (Supplementary Methods). We demonstrated that both components contributed to a model optimized for survival separation across TCGA, with the functional component carrying greater weight, whereas the immune component was the main determinant for an equivalent model in the immunotherapy-treated NSCLC cohort (Fig. 4e).

**Extended Data Fig. 10:**
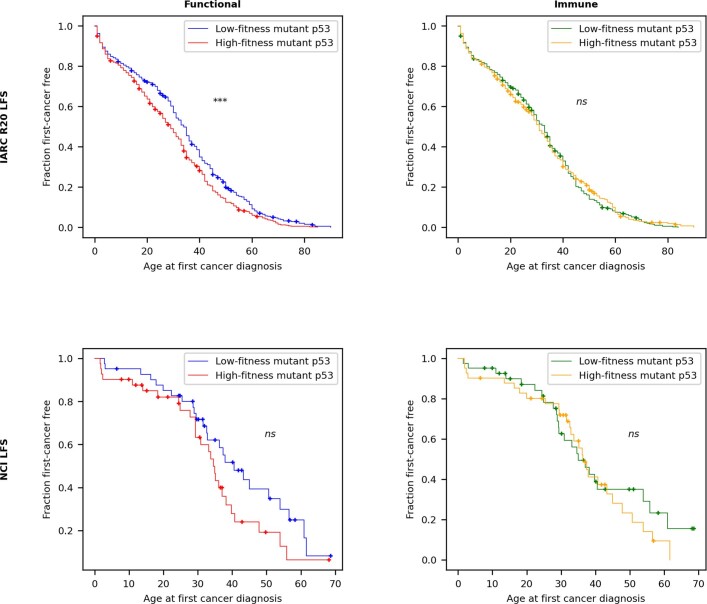
Relationships of germline mutant p53 fitness and age of tumour onset. Kaplan-Meier curves separated by median functional and immune mutant p53 fitness for first-cancer age of onset in the LFS IARC R20 germline dataset (N = 998) and the NCI LFS cohort (N = 82). Mutant p53 fitness was determined using TCGA-derived tissue-specific mutant p53 concentrations for both datasets, with individual HLA-I types for the NCI cohort and averages taken over TCGA haplotypes for the IARC dataset, which lacked individual HLA-I types.

Because germline *TP53* mutations are the primary cause of Li–Fraumeni syndrome (LFS), which is a highly cancer-prone autosomal dominant disorder^28^, we theorized that mutant p53 fitness relates to the time to first tumour formation in patients with LFS. We plotted Kaplan–Meier curves showing the age of tumour onset for persons with germline missense *TP53* mutations in the International Agency for Research on Cancer (IARC) R20 germline dataset and for an independent LFS cohort coordinated by the National Cancer Institute (NCI)^29^, stratified on the basis of mutant p53 fitness (Supplementary Methods). We found that functional and immune components were required for significant separation of patients based on time to onset, with the immune component required across a range of relative weights (Fig. 4a, b and Extended Data Fig. 10). These results may seem counterintuitive in that mutant p53 may be interpreted as ‘self’ by the adaptive immune system in patients with LFS. However, increased mutant p53 abundance, compounded by additional somatic mutations, may increase tumour immune surveillance and mutant p53 antigenicity during tumorigenesis. These findings suggest a possible role for immune surveillance and the potential for immune intervention in germline *TP53*-mutant tumours.

Finally, non-cancerous cells in diverse tissues harbour somatic *TP53* mutations that confer a competitive advantage, predisposing the clones containing such mutations to develop into cancer^30^. We collated mutation data from multiple published works across many mutated tissues (Supplementary Information) and found the same cancer hotspots in non-neoplastic cells (Fig. 4c). Unexpectedly, however, the frequency of the hotspot mutations was different. R175H was markedly under-represented in non-neoplastic cells compared with tumours (*P* < 0.0001, two-sided binomial test), whereas the potentially more immunogenic R248Q/W mutations were among the most frequent. The addition of an immune component in the non-neoplastic setting improved predictions to a substantially lower degree than in the neoplastic setting (Fig. 4d and Supplementary Table 5), supporting the hypothesis that the difference in hotspot frequency between non-cancerous and cancerous datasets is driven by the hotspot mutation’s immune fitness. We then split the non-neoplastic *TP53* mutation dataset into the largest tissue-specific subgroups and found that immune weight depended on the tissue type (Fig. 4d), although the weight was always weaker than the optimal value for fitting the TCGA mutation distribution. Overall, these findings suggest that more functionally fit mutations probably predominate in non-cancerous and precancerous lesions owing to their selective replicative advantage; for cancer to form, however, immune escape becomes critical (Fig. 4f).

We present a general mathematical framework for predicting the fitness of tumour driver mutations. For p53, we used a free fitness model that integrates the background mutation rate, protein concentration, functional fitness advantage and immune fitness cost. Hotspots were predicted to fall on a near-optimal Pareto front, with trade-offs constraining driver mutations from completely evading immune selection, as has been shown for specific hotspot mutations^31–33^. Immune fitness has less of a role in predicting the distribution of non-cancerous *TP53* mutations, which is consistent with recent observations that immune editing is less relevant in precancerous lesions^34^. Our insights therefore help define a window of opportunity for prophylactic immune intervention against mutant p53. Additionally, our model shows that mutant p53 fitness may have a role in determining the age of tumour onset in LFS, implying a benefit in targeting germline *TP53* mutations immunotherapeutically. Inducing prophylactic immunity against mutant p53 seems to be possible according to our in vitro data showing the possibility of inducing anti-mutant p53 T cell responses in healthy individuals and even against poorly immunogenic mutations when sufficient antigen concentration and proper immune co-stimulation are delivered. Our approach captures critical mechanistic determinants of mutant p53 fitness and is amenable to extensions as data become available. For instance, although we considered only functional alterations for a set of canonical p53-regulated genes in this study, future models can include additional new measures for describing mutant gain of function, such as novel binding interactions between mutant p53 and other molecules due to changes in protein conformation or concentration. Similarly, other functions reflecting the vital role of p53 as a central transcription factor may be incorporated with additional data, such as induction of apoptosis at the mitochondria, immune regulation and surveillance of transposons and other genome parasites. The latter evolutionary role of p53 in preserving genome integrity may be responsible for p53’s centrality as a bottleneck across transcriptional networks^35–37^. Finally, our free fitness framework lends itself naturally to interpretable, free energy-based machine learning models^38^, which broadens the applicability of our approach to additional topics and modalities. By quantifying the underlying mechanisms of driver mutation fitness, we can therefore uncover both fundamental knowledge about tumour evolution and new opportunities for precision therapies.

## Methods

All research involving human participants was approved by the authors’ institutional review board (MSKCC IRB), and all clinical investigation was conducted according to the principles expressed in the Declaration of Helsinki. Written informed consent was obtained from the participants.

### Reporting summary

Further information on research design is available in the Nature Research Reporting Summary linked to this paper.

## Online content

Any methods, additional references, Nature Research reporting summaries, source data, extended data, supplementary information, acknowledgements, peer review information; details of author contributions and competing interests; and statements of data and code availability are available at 10.1038/s41586-022-04696-z.

## Supplementary information

Supplementary Information This file contains Table 1, Supplementary Figs. 1–5 and Supplementary References.

Reporting Summary

Supplementary Methods

Supplementary Table 1

Supplementary Table 2

Supplementary Table 3

Supplementary Table 4

Supplementary Table 5

Supplementary Table 6

Supplementary Table 7

## Data Availability

Original data required for running the fitness model are available at https://github.com/dfhoyosg/p53_fitness_tradeoff.

## Code availability

Original code required for running the fitness model is available at https://github.com/dfhoyosg/p53_fitness_tradeoff.
