# Author Correction: Fundamental immune–oncogenicity trade-offs define driver mutation fitness

**DOI:** 10.1038/s41586-022-04879-8

**Published:** 2022-05-31

**Authors:** David Hoyos, Roberta Zappasodi, Isabell Schulze, Zachary Sethna, Kelvin César de Andrade, Dean F. Bajorin, Chaitanya Bandlamudi, Margaret K. Callahan, Samuel A. Funt, Sine R. Hadrup, Jeppe S. Holm, Jonathan E. Rosenberg, Sohrab P. Shah, Ignacio Vázquez-García, Britta Weigelt, Michelle Wu, Dmitriy Zamarin, Laura F. Campitelli, Edward J. Osborne, Mark Klinger, Harlan S. Robins, Payal P. Khincha, Sharon A. Savage, Vinod P. Balachandran, Jedd D. Wolchok, Matthew D. Hellmann, Taha Merghoub, Arnold J. Levine, Marta Łuksza, Benjamin D. Greenbaum

**Affiliations:** 1 Computational Oncology, Department of Epidemiology & Biostatistics, Memorial Sloan Kettering Cancer Center, New York, NY USA; 2 Swim Across America Laboratory and Ludwig Collaborative, Immunology Program, Parker Institute for Cancer Immunotherapy, Memorial Sloan Kettering Cancer Center, New York, NY USA; 3 Department of Medicine, Weill Cornell Medical College, New York, NY USA; 4 Parker Institute for Cancer Immunotherapy, Memorial Sloan Kettering Cancer Center, New York, NY USA; 5 Immunology and Microbial Pathogenesis Program, Weill Cornell Graduate School of Medical Sciences, New York, NY USA; 6 Hepatopancreatobiliary Service, Department of Surgery, Memorial Sloan Kettering Cancer Center, New York, NY USA; 7 Department of Medicine, Memorial Sloan Kettering Cancer Center, New York, NY USA; 8 Division of Cancer Epidemiology and Genetics, Clinical Genetics Branch, National Cancer Institute, National Institutes of Health, Rockville, MD USA; 9 Department of Pathology and Laboratory Medicine, Memorial Sloan Kettering Cancer Center, New York, NY USA; 10 Kravis Center for Molecular Oncology, Memorial Sloan Kettering Cancer Center, New York, NY USA; 11 Experimental and Translational Immunology, Health Technology, Technical University of Denmark, Lyngby, Denmark; 12 Physiology, Biophysics & Systems Biology, Weill Cornell Medicine, Weill Cornell Medical College, New York, NY USA; 13 Department of Surgery, Memorial Sloan Kettering Cancer Center, New York, NY USA; 14 Adaptive Biotechnologies, Seattle, WA USA; 15 David M. Rubenstein Center for Pancreatic Cancer Research, Memorial Sloan Kettering Cancer Center, New York, NY USA; 16 Human Oncology and Pathogenesis Program, Memorial Sloan Kettering Cancer Center, New York, NY USA; 17 Thoracic Oncology Service, Memorial Sloan Kettering Cancer Center, New York, NY USA; 18 Simons Center for Systems Biology, Institute for Advanced Study, Princeton, NJ USA; 19 Department of Oncological Sciences, Tisch Cancer Institute, Icahn School of Medicine at Mount Sinai, New York, NY USA

**Keywords:** Tumour immunology, Tumour-suppressor proteins, Computational models, Evolutionary theory

Correction to: *Nature* 10.1038/s41586-022-4696-z,published online 11 May 2022.

In the version of this article initially published, the surname of author Chaitanya Bandlamudi was misspelt as Bandlamundi. The error has been corrected in the HTML and PDF versions of the article

