# Supplementary material for: Fundamental immune–oncogenicity trade-offs define driver mutation fitness
Source: Nature. 2022 May 11;606(7912):172–9. doi: 10.1038/s41586-022-04696-z (PMC9159948; doi:10.1038/s41586-022-04696-z)
Supplement: Supplementary file 1 — This file contains Table 1, Supplementary Figs. 1–5 and Supplementary References. [file 41586_2022_4696_MOESM1_ESM.pdf]

nature portfolio

https://doi.org/10.1038/s41586-022-04696-z

Supplementary information

Fundamental immune–oncogenicity trade-offs define driver mutation fitness

In the format provided by the authors and unedited

Supplementary Information

Selection of representative cancer driver genes and hotspots. We selected a total of 26 representative tumor suppressors and oncogenes implicated in driving tumorigenesis and commonly mutated in TCGA[1,54]. These genes are: *KRAS*, *HRAS*, *NRAS*, *PTEN*, *PIK3CA*, *PIK3R1*, *EGFR*, *BRAF*, *NOTCH1*, *RB1*, *ARID1A*, *MYC*, *POLE*, *MLH1*, *MSH2*, *IDH1*, *CDKN2A*, *CTNNB1*, *ERBB2*, *SMAD2*, *SMAD4*, *APC*, *BRCA1*, *BRCA2*, *FAT4*, and *TP53*. We only considered missense mutations, which are amenable to our model predictions since, for example, there are fewer doubts concerning mutant protein expression. We manually curated hotspots from TCGA. The genes and their hotspots are shown in Table 1.

Gene	Hotspots
KRAS	G12D, G12V, G12C
HRAS	Q61R
NRAS	Q61R, Q61K
PTEN	R130Q, R130G
APC	S2307L
PIK3CA	E545K, H1047R, E542K
PIK3R1	G376R, N564D
EGFR	L858R, A289V, G598V
BRCA1	E1258D
BRCA2	A1393V, E3342K
BRAF	V600E
NOTCH1	A465T
RB1	R876C, R741C, R451C
ARID1A	G2087R
MYC	S161L
POLE	P286R, V411L
MLH1	R265C, R385C
MSH2	R929Q, R406Q, K871N
IDH1	R132H, R132C
ARF	H83Y, P114L, D108Y
CTNNB1	S37F, T41A, S45P
ERBB2	S310F
SMAD2	R120Q, S276L, R321Q
SMAD4	R361H, R361C
FAT4	R2685Q, R1671C, D1790N, H2514Y
TP53	R175H, R248Q, R273H, R248W, R273C, R282W, G245S, Y220C

Table 1: Selected cancer driver genes and hotspots.

Selection of representative genes and mutations implicated in non-cancer diseases. We tested if mutations which are less conserved are more likely to generate more immunogenic peptides (as defined by likelihood to be presented on class I MHC), outside of the cancer setting. To do so, we examined dozens of genes which have single nucleotide polymorphisms that are associated with non-cancerous diseases. We filtered out any gene for which there was at least some evidence that it had functional importance for cancer development, or whose symptoms manifested as benign tumors. We kept genes in which, to

date, mutations only have strong documented evidence for roles in non-cancerous diseases.

We considered a total of nine genes. Five of these genes are hemoglobin subunits (*HBA*, *HBB*, *HBD*, *HG1*, *HG2*), and the other four are related to other non-cancer associated conditions (*PAH*, *F8*, *PHEX*, *POGZ*). Mutations in hemoglobin subunits are well-documented, mainly the *HBA* and *HBB* subunits which are the major hemoglobin subunits in adults[86, 87]. While some mutations are benign and do not alter hemoglobin function or stability, there are multiple mutations which are functionally destructive. Mutations in phenylalanine hydroxylase (*PAH*) are associated with phenylketonuria, resulting in reduced phenylalanine metabolism[88]. Mutations in Factor VIII (*F8*) contribute to hemophilia A[89]. Mutations in phosphate-regulating neutral endopeptidase, X-linked (*PHEX*) are related to bone deformations due to inhibited phosphate retention[90]. Mutations in the pogo transposable element with ZNF domain (*POGZ*) gene are related to White-Sutton syndrome[91]. In all cases, mutations within the genes in question may have a spectrum of functional effects, from negligible changes to significant alterations in function or protein stability.

We collated single-nucleotide polymorphism data for these genes available from the NCBI's dbSNP[92] and mapped genomic mutations to amino acid alterations using the GRCh38 reference genome, identifying a total of 2,195 missense mutations across these 9 genes. We then only kept the mutation set which were unequivocally not-pathogenic (annotated as "benign", "protective", "likely-benign", and/or "benign-likely-benign") or pathogenic (annotated as "pathogenic", "likely-pathogenic", and/or "pathogenic-likely-pathogenic") as determined by the NCBI's ClinVar annotation system[93]. This resulted in 113 not-pathogenic mutations and 836 pathogenic mutations for a total of 949 mutations. All other mutations were not considered for the analysis.

For each gene, we compared inferred population-averaged likelihood of class-I MHC presentation for the nine 9-mer peptides surrounding the mutation across the "non-pathogenic" (i.e., more sequence conservation) and "pathogenic" (i.e., poor sequence conservation) groups.

Mutation datasets. Our models are applied to somatic mutations across commonly mutated tumor suppressors and oncogenes, as well as pre-neoplastic *TP53* mutations. For mutant *TP53*, we train the mutation model on somatic TCGA *TP53* mutation distributions downloaded from the Genomic Data Commons[54]. We consider a total of 2,764 p53 mutations across 2,580 tumors in TCGA. We only consider missense mutations which arise from a single-nucleotide variation.

In examining models without concentration for all considered commonly mutated tumor suppressors and oncogenes, we utilized missense mutation distributions from both COSMIC (version 90)[53] and TCGA, as available from the Genomic Data Commons[54]. When comparing COSMIC and TCGA, we filtered out the mutations from COSMIC that also appear in TCGA. When comparing TCGA and IARC, we filtered out the mutations from IARC that also appear in TCGA. We only considered missense mutations from single-nucleotide variations to limit confounding issues with protein expression in other types of mutants, such as truncation mutants. Where possible, we assured that we considered properly matched primary canonical transcripts of these genes across databases. For *KRAS*, where there are two well-expressed isoforms which have largely conserved amino acid sequences, we focused on isoform "A", which is listed as the canonical transcript in the UniProt database[94]. For *TP53*, we excluded all mutations at codons 72 and 46 involving proline/arginine or proline/serine, respectively, as these are well-known polymorphisms.

It has become clear in recent years that *TP53* mutations exist in cells which are non-cancerous[30]. To date, there is no large-scale non-tumor somatic p53 mutation database which collates data from multi-

ple sources, such as IARC does for p53 mutations in tumors and in patients with Li-Fraumeni Syndrome. To address this, we assembled SNV-generated missense *TP53* mutations in non-tumor tissues across 17 publications into one non-neoplastic *TP53* mutation database, collating 3,541 missense mutation occurrences (3,135 of which are in the DNA binding domain, defined here as amino acids [100, 300]), comparable in order-of-magnitude to other databases such as IARC R20 Europe (N=7,579) and TCGA (N=2,764). We gathered mutations in the blood from eight datasets[95–100], urothelium mutations in one dataset[101], bladder mutations in one dataset[102], bronchial mutations in three datasets[103–105], colorectal mutations in three datasets[106–108], gynecological mutations in seven datasets[109–115], esophageal mutations in nine datasets[116–124], liver mutations in one dataset[125], skin mutations in ten datasets[126–134], and four pan-tissue datasets[135–138]. In all cases we assured that only mutations which were identified as not being cancer-derived were included.

For LFS mutations in IARC, we used the R20 version of the IARC germline database[49]. We excluded all data which may have been contributed by the NCI, in order to avoid analyzing survival for the same person twice. We only considered missense mutations.

Kaplan-Meier Curves. We examined the role of inferred mutant p53 functional, immune, and total fitness on survival in both non-immunotherapy treated (TCGA, pan-cancer) and immune checkpoint-blockade (ICB)-treated (non-small cell lung cancer, Memorial Sloan Kettering Cancer Center (MSKCC)) cohorts. For the IARC R20 Li-Fraumeni patients with germline *TP53* mutations, we plotted a Kaplan-Meier curve for first age of onset of a tumor. In all cases we estimated the mutant fitness using the inferred tissue-specific concentration and the matched haplotype where possible. We used the matched mutant and haplotype for defining the immune fitness for all cohorts except for the IARC R20 Li-Fraumeni cohort. For the IARC Li-Fraumeni cohort, we infer the haplotype using TCGA haplotype distribution.

Description of statistical methods. We used Welch's T-test and the Mann-Whitney U-test for categorical tests. We used the Pearson and the Spearman correlations for continuous variables. For model training and testing, we calculated the Kullback-Leibler divergence using the observed and predicted mutation frequencies. The confidence intervals in SI Fig. 2 are 95% confidence intervals computed using the normal approximation. The log-rank test is used for testing separation significance in Kaplan-Meier curves.

Supplementary Figures

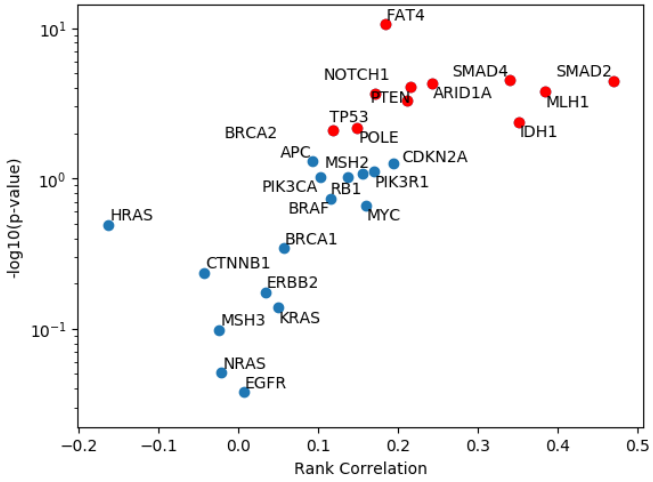

Supplementary Figure 1 | Correlation of observed mutation frequencies to expected intrinsic background mutation frequencies.

Comparison of the expected background dinucleotide mutation frequencies and the observed mutation frequencies of selected cancer driver genes in TCGA.

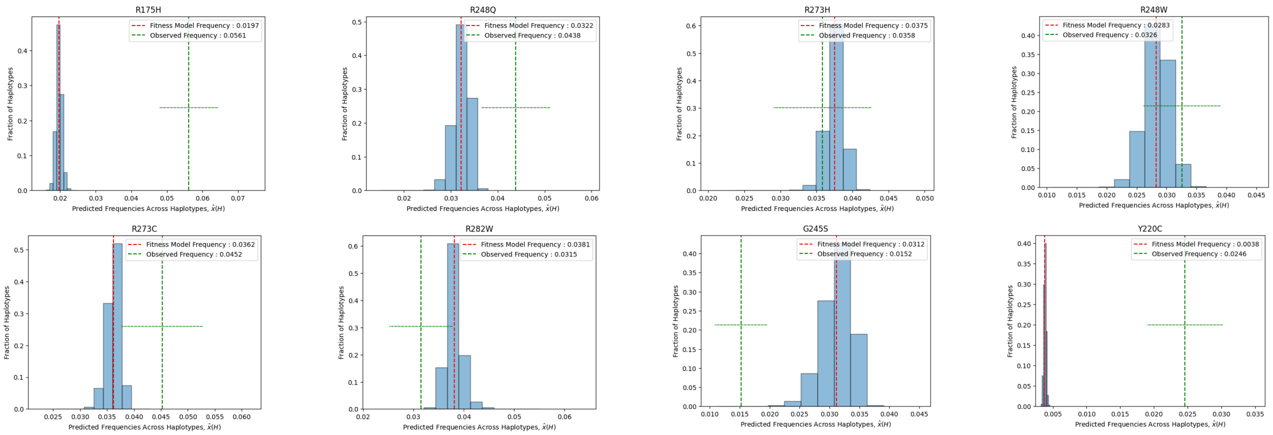

Supplementary Figure 2 | Additional fitness model results on specific hotspots.
Distributions of predicted HLA-I haplotype-specific frequency values for each of the hotspot mutations for the TCGA pan-cancer model. The distributions are computed across haplotypes of patients in TCGA, where different HLA-I haplotypes correspond to different levels of immune selection. The HLA-I haplotype averaged frequencies are marked with dashed red lines, the observed frequencies are marked with vertical dashed green lines, and the horizontal dashed green lines correspond to 95% confidence intervals of the observed mutation frequency.

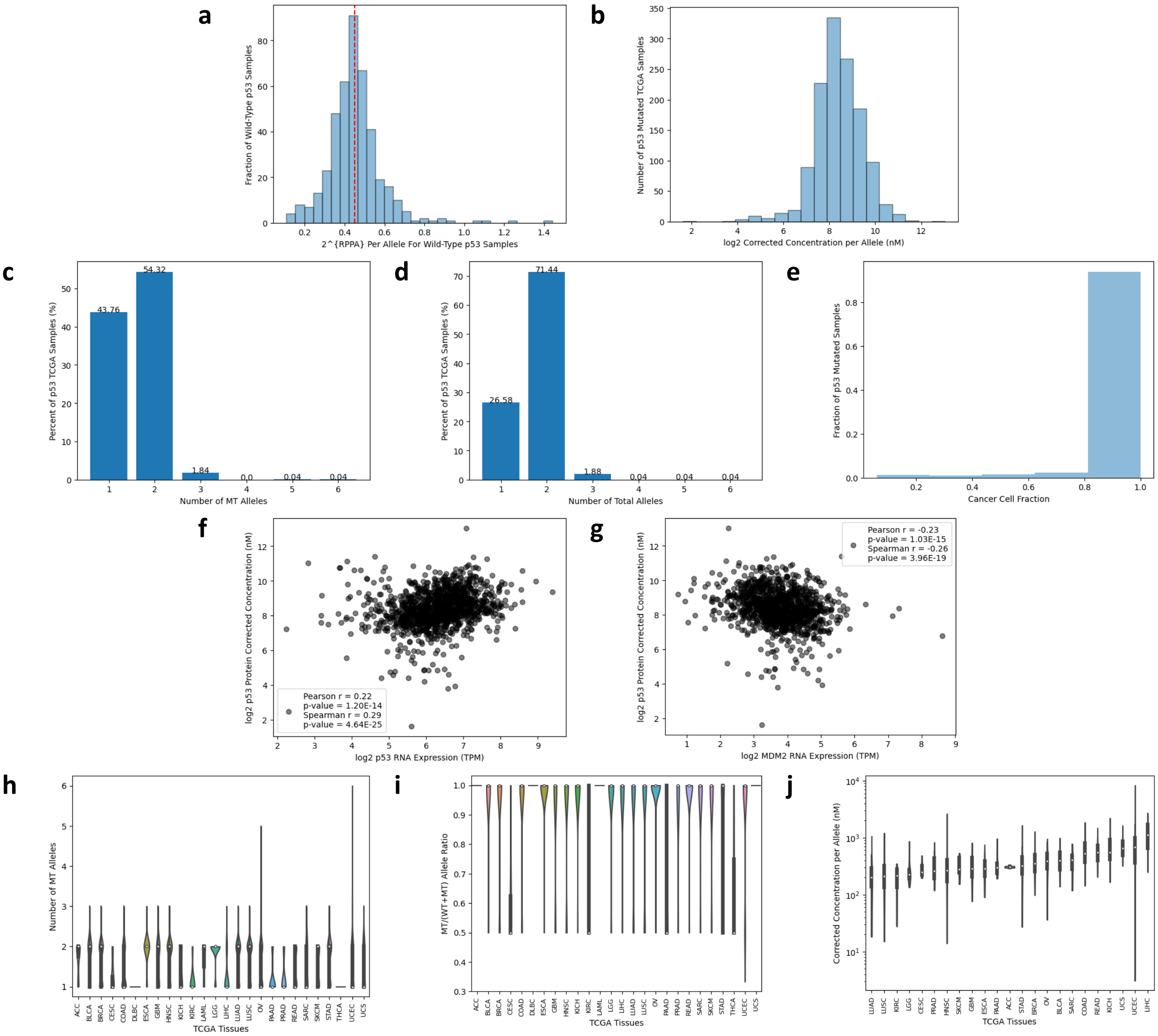

Supplementary Figure 3 | Heterogeneity and inferred mutant p53 concentration.
a, Distribution of wild-type p53 concentration used for transforming RPPA values to concentration values. **b,** Distribution of mutant p53 concentration across mutations and tissues. **c-d,** Distribution of MT and total number of *TP53* alleles across TCGA. **e,** Cancer cell fraction distribution of *TP53* mutations. **f-g,** Relationships between *TP53* and *MDM2* RNA and inferred p53 protein expression. **h-i,** Distribution of mutant and fraction of mutant alleles across different TCGA tissues. **j,** Distribution of inferred mutant p53 concentration across TCGA tissues.

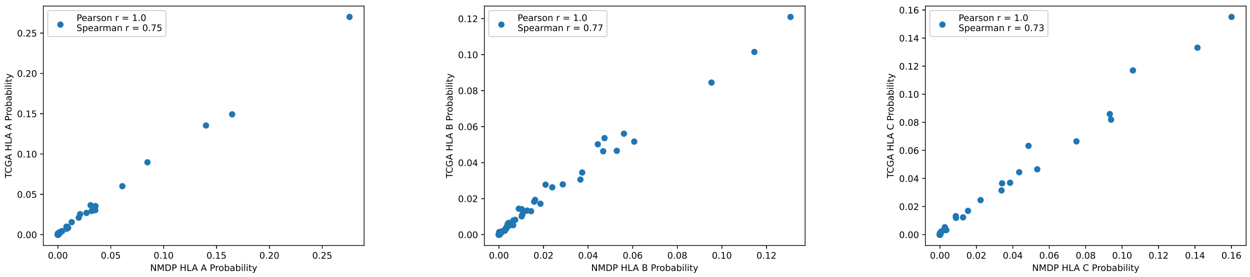

Supplementary Figure 4 | Relationships between haplotype populations.
Highly-correlated shared HLA-I frequencies in simulated and TCGA MHC-I haplotype populations.

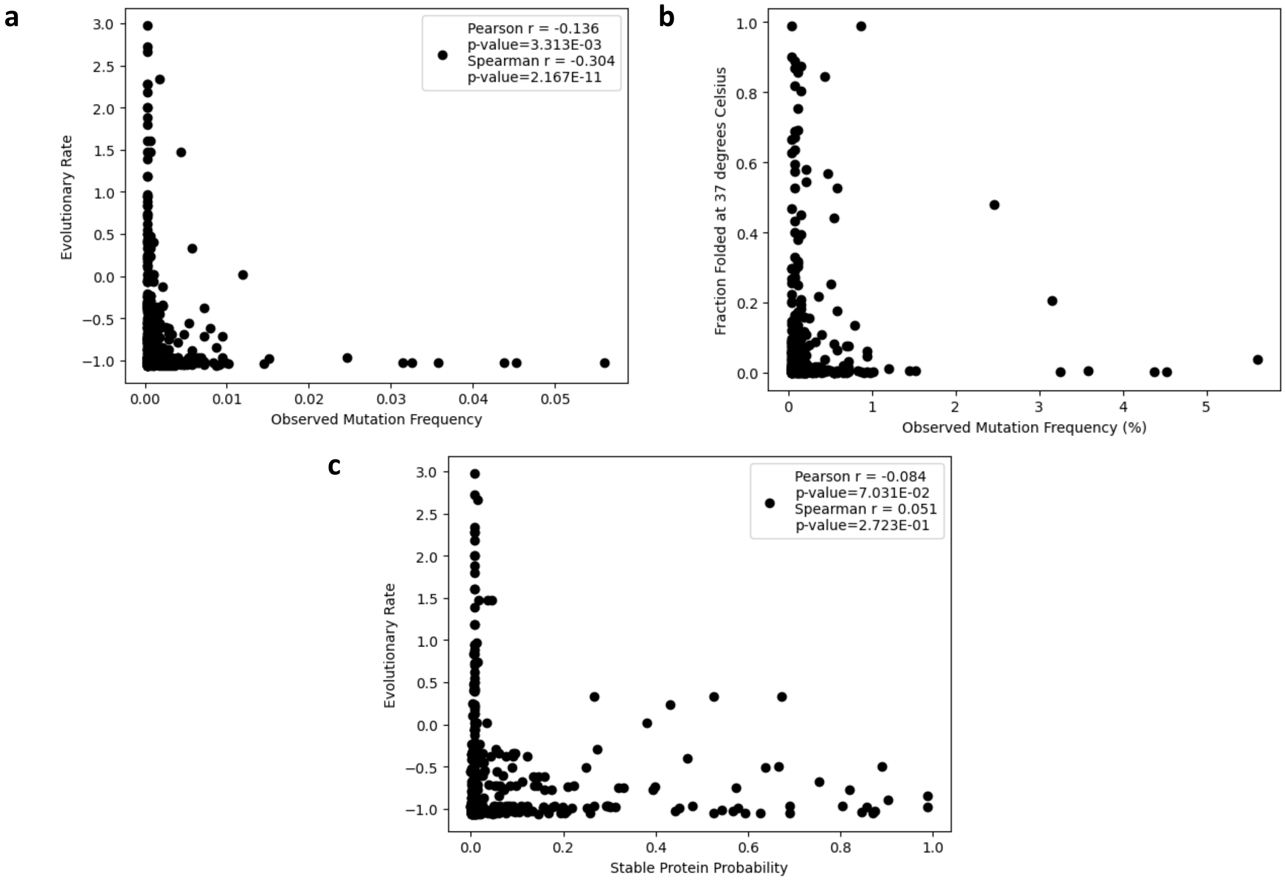

Supplementary Figure 5 | Relationships between inferred mutant p53 conservation, stability, and mutation frequency in additional models.

a-b, Relationship between conservation, stability and mutation frequency. Most hotspots are conserved and induce protein instability. The temperature used for the stability calculations is 310 K, approximately human body temperature. **c,** Relationship between conservation and protein stability.

References

86. Thom, C. S., Dickson, C. F., Gell, D. A. & Weiss, M. J. Hemoglobin variants: biochemical properties and clinical correlates. *Cold Spring Harbor Perspectives in Medicine* **3**, a011858 (2013).

87. Kaufman, D. P., Khattar, J. & Lappin, S. L. Physiology, Fetal Hemoglobin. *StatPearls [Internet]* (2021).

88. Scriver, C. R. The *PAH* gene, phenylketonuria, and a paradigm shift. *Human Mutation* **28**, 831–845 (2007).

89. Oldenburg, J. & El-Maarri, O. New insight into the molecular basis of hemophilia A. *International Journal of Hematology* **83**, 96–102 (2006).

90. Dixon, P. H. *et al.* Mutational analysis of *PHEX* gene in X-linked hypophosphatemia. *The Journal of Clinical Endocrinology & Metabolism* **83**, 3615–3623 (1998).

91. Assia Batzir, N. *et al.* Phenotypic expansion of *POGZ*-related intellectual disability syndrome (White-Sutton syndrome). *American Journal of Medical Genetics Part A* **182**, 38–52 (2020).

92. Sherry, S. T. *et al.* dbSNP: the NCBI database of genetic variation. *Nucleic Acids Research* **29**, 308–311 (2001).

93. Landrum, M. J. *et al.* ClinVar: improving access to variant interpretations and supporting evidence. *Nucleic Acids Research* **46**, D1062–D1067 (2018).

94. Consortium, T. U. UniProt: The universal protein knowledgebase in 2021. *Nucleic Acids Research* **49**, D480–D489 (2021).

95. Abelson, S. *et al.* Prediction of acute myeloid leukaemia risk in healthy individuals. *Nature* **559**, 400–404 (2018).

96. Desai, P. *et al.* Somatic mutations precede acute myeloid leukemia years before diagnosis. *Nature Medicine* **24**, 1015–1023 (2018).

97. Wong, T. N. *et al.* Role of *TP53* mutations in the origin and evolution of therapy-related acute myeloid leukaemia. *Nature* **518**, 552–555 (2015).

98. Jaiswal, S. *et al.* Age-related clonal hematopoiesis associated with adverse outcomes. *New England Journal of Medicine* **371**, 2488–2498 (2014).

99. Genovese, G. *et al.* Clonal hematopoiesis and blood-cancer risk inferred from blood DNA sequence. *New England Journal of Medicine* **371**, 2477–2487 (2014).

100. Xie, M. *et al.* Age-related mutations associated with clonal hematopoietic expansion and malignancies. *Nature Medicine* **20**, 1472–1478 (2014).

101. Li, R. *et al.* Macroscopic somatic clonal expansion in morphologically normal human urothelium. *Science* **370**, 82–89 (2020).

102. Lawson, A. R. *et al.* Extensive heterogeneity in somatic mutation and selection in the human bladder. *Science* **370**, 75–82 (2020).

103. Yoshida, K. *et al.* Tobacco smoking and somatic mutations in human bronchial epithelium. *Nature* **578**, 266–272 (2020).

104. Franklin, W. A. *et al.* Widely dispersed p53 mutation in respiratory epithelium. a novel mechanism for field carcinogenesis. *The Journal of Clinical Investigation* **100**, 2133–2137 (1997).

105. Kadara, H. *et al.* Driver mutations in normal airway epithelium elucidate spatiotemporal resolution of lung cancer. *American Journal of Respiratory and Critical Care Medicine* **200**, 742–750 (2019).

106. Lee-Six, H. *et al.* The landscape of somatic mutation in normal colorectal epithelial cells. *Nature* **574**, 532–537 (2019).

107. Olafsson, S. *et al.* Somatic evolution in non-neoplastic IBD-affected colon. *Cell* **182**, 672–684 (2020).

108. Matas, J. *et al.* Colorectal cancer is associated with the presence of cancer driver mutations in normal colon. *medRxiv* (2021).

109. Salk, J. J. *et al.* Ultra-sensitive *TP53* sequencing for cancer detection reveals progressive clonal selection in normal tissue over a century of human lifespan. *Cell Reports* **28**, 132–144 (2019).

110. Krimmel, J. D. *et al.* Ultra-deep sequencing detects ovarian cancer cells in peritoneal fluid and reveals somatic *TP53* mutations in noncancerous tissues. *Proceedings of the National Academy of Sciences* **113**, 6005–6010 (2016).

111. Krimmel-Morrison, J. D. *et al.* Characterization of *TP53* mutations in Pap test DNA of women with and without serous ovarian carcinoma. *Gynecologic Oncology* **156**, 407–414 (2020).

112. Paracchini, L. *et al.* Detection of *TP53* clonal variants in Papanicolaou test samples collected up to 6 years prior to high-grade serous epithelial ovarian cancer diagnosis. *JAMA Network Open* **3**, e207566–e207566 (2020).

113. Jia, L. *et al.* Endometrial glandular dysplasia with frequent p53 gene mutation: a genetic evidence supporting its precancer nature for endometrial serous carcinoma. *Clinical Cancer Research* **14**, 2263–2269 (2008).

114. Moore, L. *et al.* The mutational landscape of normal human endometrial epithelium. *Nature* **580**, 640–646 (2020).

115. Anglesio, M. S. *et al.* Cancer-associated mutations in endometriosis without cancer. *New England Journal of Medicine* **376**, 1835–1848 (2017).

116. Martincorena, I. *et al.* Somatic mutant clones colonize the human esophagus with age. *Science* **362**, 911–917 (2018).

117. Yokoyama, A. *et al.* Age-related remodelling of oesophageal epithelia by mutated cancer drivers. *Nature* **565**, 312–317 (2019).

118. Prevo, L. J., Sanchez, C. A., Galipeau, P. C. & Reid, B. J. p53-mutant clones and field effects in Barrett's esophagus. *Cancer Research* **59**, 4784–4787 (1999).

119. Mandard, A.-M., Hainaut, P. & Hollstein, M. Genetic steps in the development of squamous cell carcinoma of the esophagus. *Mutation Research/Reviews in Mutation Research* **462**, 335–342 (2000).

120. Weaver, J. M. *et al.* Ordering of mutations in preinvasive disease stages of esophageal carcinogenesis. *Nature Genetics* **46**, 837–843 (2014).

121. Waridel, F. *et al.* Field cancerisation and polyclonal p53 mutation in the upper aero-digestive tract. *Oncogene* **14**, 163–169 (1997).

122. Ross-Innes, C. S. *et al.* Whole-genome sequencing provides new insights into the clonal architecture of Barrett's esophagus and esophageal adenocarcinoma. *Nature Genetics* **47**, 1038–1046 (2015).

123. Stachler, M. D. *et al.* Paired exome analysis of Barrett's esophagus and adenocarcinoma. *Nature Genetics* **47**, 1047–1055 (2015).

124. Yuan, W. *et al.* Clonal evolution of esophageal squamous cell carcinoma from normal mucosa to primary tumor and metastases. *Carcinogenesis* **40**, 1445–1451 (2019).

125. Kim, S. K. *et al.* Comprehensive analysis of genetic aberrations linked to tumorigenesis in regenerative nodules of liver cirrhosis. *Journal of Gastroenterology* **54**, 628–640 (2019).

126. Ling, G. *et al.* Persistent p53 mutations in single cells from normal human skin. *The American Journal of Pathology* **159**, 1247–1253 (2001).

127. Martincorena, I. *et al.* High burden and pervasive positive selection of somatic mutations in normal human skin. *Science* **348**, 880–886 (2015).

128. Jonason, A. S. *et al.* Frequent clones of p53-mutated keratinocytes in normal human skin. *Proceedings of the National Academy of Sciences* **93**, 14025–14029 (1996).

129. Simons, B. D. Deep sequencing as a probe of normal stem cell fate and preneoplasia in human epidermis. *Proceedings of the National Academy of Sciences* **113**, 128–133 (2016).

130. Ren, Z.-P. *et al.* Benign clonal keratinocyte patches with p53 mutations show no genetic link to synchronous squamous cell precancer or cancer in human skin. *The American Journal of Pathology* **150**, 1791 (1997).

131. Bäckvall, H. *et al.* Mutation spectra of epidermal p53 clones adjacent to basal cell carcinoma and squamous cell carcinoma. *Experimental Dermatology* **13**, 643–650 (2004).

132. Hernando, B. *et al.* The effect of age on the acquisition and selection of cancer driver mutations in sun-exposed normal skin. *Annals of Oncology* **32**, 412–421 (2021).

133. Tang, J. *et al.* The genomic landscapes of individual melanocytes from human skin. *Nature* **586**, 600–605 (2020).

134. Muradova, E. *et al.* Noninvasive assessment of epidermal genomic markers of UV exposure in skin. *Journal of Investigative Dermatology* **141**, 124–131 (2021).

135. Coorens, T. H. *et al.* Extensive phylogenies of human development inferred from somatic mutations. *Nature* **597**, 387–392 (2021).

136. Moore, L. *et al.* The mutational landscape of human somatic and germline cells. *Nature* **597**, 381–386 (2021).

137. Xia, L. *et al.* Statistical analysis of mutant allele frequency level of circulating cell-free DNA and blood cells in healthy individuals. *Scientific Reports* **7**, 1–7 (2017).

138. Yizhak, K. *et al.* RNA sequence analysis reveals macroscopic somatic clonal expansion across normal tissues. *Science* **364** (2019).
