# Supplementary material for: Fundamental immune–oncogenicity trade-offs define driver mutation fitness
Source: Nature. 2022 May 11;606(7912):172–9. doi: 10.1038/s41586-022-04696-z (PMC9159948; doi:10.1038/s41586-022-04696-z)
Supplement: Supplementary file 2 — Reporting Summary [file 41586_2022_4696_MOESM2_ESM.pdf]

nature portfolio

Corresponding author(s): Roberta Zappasodi, Taha Merghoub, and Benjamin D. Greenbaum

Last updated by author(s): March 4, 2022

Reporting Summary

Nature Portfolio wishes to improve the reproducibility of the work that we publish. This form provides structure for consistency and transparency in reporting. For further information on Nature Portfolio policies, see our Editorial Policies and the Editorial Policy Checklist.

Statistics

For all statistical analyses, confirm that the following items are present in the figure legend, table legend, main text, or Methods section.

n/a	Confirmed	
☐	☒	The exact sample size (*n*) for each experimental group/condition, given as a discrete number and unit of measurement
☐	☒	A statement on whether measurements were taken from distinct samples or whether the same sample was measured repeatedly
☐	☒	The statistical test(s) used AND whether they are one- or two-sided<br>*Only common tests should be described solely by name; describe more complex techniques in the Methods section.*
☐	☒	A description of all covariates tested
☐	☒	A description of any assumptions or corrections, such as tests of normality and adjustment for multiple comparisons
☐	☒	A full description of the statistical parameters including central tendency (e.g. means) or other basic estimates (e.g. regression coefficient) AND variation (e.g. standard deviation) or associated estimates of uncertainty (e.g. confidence intervals)
☐	☒	For null hypothesis testing, the test statistic (e.g. *F*, *t*, *r*) with confidence intervals, effect sizes, degrees of freedom and *P* value noted<br>*Give P values as exact values whenever suitable.*
☐	☒	For Bayesian analysis, information on the choice of priors and Markov chain Monte Carlo settings
☒	☐	For hierarchical and complex designs, identification of the appropriate level for tests and full reporting of outcomes
☐	☒	Estimates of effect sizes (e.g. Cohen's *d*, Pearson's *r*), indicating how they were calculated

Our web collection on statistics for biologists contains articles on many of the points above.

Software and code

Policy information about availability of computer code

Data collection	No software was used to collect data.
Data analysis	Statistical analysis and figures were generated with Python 3. Peptide:HLA-I presentation was inferred in silico using the NetMHC 3.4, NetMHC 4.0, and NetMHCpan 4.1 software. Amino acid conservation was inferred from the ConSurf server and with hmmer 3.3.2, cdhit 4.8.1, MAFFT 7.475, and rate4site 3.0.0. Experimental data was obtain from a 4 laser Aurora full spectrum cytometer (UV-V-B-R, Cytek) and analyzed using the FlowJo software version 10.7.1. Custom code for the fitness model is available at: https://github.com/dfhoyosg/p53_fitness_tradeoff.

For manuscripts utilizing custom algorithms or software that are central to the research but not yet described in published literature, software must be made available to editors and reviewers. We strongly encourage code deposition in a community repository (e.g. GitHub). See the Nature Portfolio guidelines for submitting code & software for further information.

Data

Policy information about availability of data

All manuscripts must include a data availability statement. This statement should provide the following information, where applicable:

- Accession codes, unique identifiers, or web links for publicly available datasets
- A description of any restrictions on data availability
- For clinical datasets or third party data, please ensure that the statement adheres to our policy

The IARC R20 somatic and germline mutation datasets as well as the p53 missense mutation functional information can be downloaded from: https://tp53.isb-cgc.org/.
The somatic p53 non-neoplastic mutations were derived from the referenced publications.
KRAS hotspot functional information derived from the referenced publication.

All TCGA data except RPPA information was downloaded from the Genomic Data Commons: portal.gdc.cancer.gov.
RPPA information was downloaded from the TCPA portal: tcpaportal.org/tcpa/download.html.
The gene DNA sequences were downloaded from: https://www.ncbi.nlm.nih.gov.
The gene protein product sequences were downloaded from UniProt: https://www.uniprot.org.
COSMIC mutation frequencies were downloaded from COSMIC: cancer.sanger.ac.uk/cosmic.
Simulated HLA-I haplotypes were derived using HLA-I frequencies derived from the National Marrow Donor Program: allelefrequencies.net.
Original data required for running the fitness model is available at: https://github.com/dfhoyosg/p53_fitness_tradeoff.

Field-specific reporting

Please select the one below that is the best fit for your research. If you are not sure, read the appropriate sections before making your selection.

☒ Life sciences ☐ Behavioural & social sciences ☐ Ecological, evolutionary & environmental sciences

For a reference copy of the document with all sections, see nature.com/documents/nr-reporting-summary-flat.pdf

Life sciences study design

All studies must disclose on these points even when the disclosure is negative.

Sample size

Sample sizes were dependent on the availability of data. In all cases, we used samples with SNV-derived missense mutations. Our framework takes into account sample size when predicting mutation frequencies and model complexity. Since we considered driver genes which are well-known to be often mutated in cancer, we deemed these sample sizes sufficient.

Data exclusions

All non-SNV-derived missense mutations were excluded from the analysis in all cases. We also excluded the well-known codon 72 and 46 p53 polymorphisms involving proline/arginine and proline/serine amino acids, respectively.

For the National Cancer Institute germline p53 mutation cohort, non-melanoma skin cancers and HPV-associated high grade dysplasias were excluded.

Replication

Antigen presentation experiments were repeated three times each for each peptide:HLA-I prediction. All repetition attempts were successful. For each repetition the results were consistent, and the standard error bars are shown.

Randomization

For experiments, two donors without a history of cancer with the HLA-A*02:01 HLA-I molecule were chosen to test presentation of antigens by this HLA-I molecule. We also inferred presentation of the examined peptides from the other present HLA-I molecules within the two donors, and found that, where prediction allowed, none of the present HLA-I molecules were able to present the tested peptides. For the MIRA experiments, random healthy donor PBMCs were utilized. Cancer samples were determined by sample availability.

Blinding

Blinding was not relevant to our study since we were interested in testing the prediction that there would be differential presentation of p53 hotspots neoantigens.

Reporting for specific materials, systems and methods

We require information from authors about some types of materials, experimental systems and methods used in many studies. Here, indicate whether each material, system or method listed is relevant to your study. If you are not sure if a list item applies to your research, read the appropriate section before selecting a response.

Materials & experimental systems

n/a	Involved in the study	
☐	☒	Antibodies
☒	☐	Eukaryotic cell lines
☒	☐	Palaeontology and archaeology
☒	☐	Animals and other organisms
☐	☒	Human research participants
☐	☒	Clinical data
☒	☐	Dual use research of concern

Methods

n/a	Involved in the study	
☒	☐	ChIP-seq
☐	☒	Flow cytometry
☒	☐	MRI-based neuroimaging

Antibodies

Antibodies used

For RPPA analysis: p53 antibody #9282 (Cell Signaling Technology). For the experiments: anti-human CD3-BUV395 (BD Biosciences, cat. no. 740283), anti-human CD4-AlexaFluor700 (Invitrogen, cat. no. 56-0047-42), anti-human CD8-AlexaFluor647 (BD Biosciences, cat. no. 557708), anti-human CD45RA-BUV737 (BD Biosciences, cat. no. 564442), anti-human CD62L-PE (BD Biosciences, cat. no. 555544), anti-human IFN-γ-FITC (Invitrogen, cat. no. BMS107FI), and anti-human Ki67-APC-eFluor 780 (Invitrogen, cat. no. 47-5698-82).

Validation

The p53 antibody #9282 (Cell Signaling Technology) has been validated using SimpleChIP® Enzymatic Chromatin IP Kits (https://

Validation	www.cellsignal.com/products/primary-antibodies/p53-antibody/9282).<br><br>For details regarding validation for the experimental antibodies, please see the relevant references on the appropriate websites:<br><br>https://www.bdbiosciences.com/us/reagents/research/antibodies-buffers/immunology-reagents/anti-human-antibodies/cell-surface-antigens/buv395-mouse-anti-human-cd3-hit3a/p/740283<br><br>https://www.thermofisher.com/antibody/product/CD4-Antibody-clone-SK3-SK-3-Monoclonal/56-0047-42<br><br>https://www.bdbiosciences.com.com/eu/reagents/research/antibodies-buffers/immunology-reagents/anti-human-antibodies/cell-surface-antigens/alexa-fluor-647-mouse-anti-human-cd8-rpa-t8/p/557708<br><br>https://www.bdbiosciences.com/us/reagents/research/antibodies-buffers/immunology-reagents/anti-human-antibodies/cell-surface-antigens/buv737-mouse-anti-human-cd45ra-hi100/p/612846<br><br>https://www.bdbiosciences.com/us/applications/research/t-cell-immunology/regulatory-t-cells/surface-markers/human/pe-mouse-anti-human-cd62l-dreg-56/p/555544<br><br>https://www.thermofisher.com/antibody/product/IFN-gamma-Antibody-clone-GZ-4-Monoclonal/BMS107FI<br><br>https://www.thermofisher.com/antibody/product/Ki-67-Antibody-clone-SolA15-Monoclonal/47-5698-82

Human research participants

Policy information about studies involving human research participants

Population characteristics	The National Cancer Institute germline p53 mutation cohort consisted of 82 individuals. As of March 24, 2020, 52 individuals had at least one cancer while 30 had remained cancer-free. Additional information is available in the Methods section. Cancer patients had ovarian and bladder cancer.
Recruitment	Participants or their legal guardians signed informed consent, completed questionnaires, and provided medical records, including pathology and genetic testing reports to validate cancer diagnoses and TP53 variant, as previously described (Mai et al., 2016, Cancer). Cancer samples were determined by availability.
Ethics oversight	The National Cancer Institute's and Memorial Sloan Kettering Cancer Center's Institutional Review Board.

Note that full information on the approval of the study protocol must also be provided in the manuscript.

Clinical data

Policy information about clinical studies

All manuscripts should comply with the ICMJE guidelines for publication of clinical research and a completed CONSORT checklist must be included with all submissions.

Clinical trial registration	NCT01443468
Study protocol	https://clinicaltrials.gov/ct2/show/NCT01443468
Data collection	Data began to be collected on September 29, 2011.
Outcomes	Age of cancer onset was the primary outcome measure used for the Kaplan-Meier survival analysis.

Flow Cytometry

Plots

Confirm that:

☒ The axis labels state the marker and fluorochrome used (e.g. CD4-FITC).

☒ The axis scales are clearly visible. Include numbers along axes only for bottom left plot of group (a 'group' is an analysis of identical markers).

☒ All plots are contour plots with outliers or pseudocolor plots.

☒ A numerical value for number of cells or percentage (with statistics) is provided.

Methodology

Sample preparation	Human peripheral mononuclear cells for in vitro assays were collected from HLA-I typed healthy donors under approved protocols. Refer to the methods for further details.
Instrument	Data was acquired on a 4 laster Aurora full spectrum cytometer (UV-V-B-R, Cytek).
Software	FlowJo software (version 10.7.1).

Cell population abundance	CD4 and CD8 cells were enriched by magnetic separation (>90% purity). Dendritic cells were differentiated from CD14+ monocytes in vitro reaching >80% purity.
Gating strategy	The gating strategy is fully displayed in Extended Data Figure 7.

☒ Tick this box to confirm that a figure exemplifying the gating strategy is provided in the Supplementary Information.
