# Supplementary material for: Fundamental immune–oncogenicity trade-offs define driver mutation fitness
Source: Nature. 2022 May 11;606(7912):172–9. doi: 10.1038/s41586-022-04696-z (PMC9159948; doi:10.1038/s41586-022-04696-z)
Supplement: Supplementary file 3 — Supplementary Methods [file 41586_2022_4696_MOESM3_ESM.pdf]

Methods

Background mutation distribution

Predicted background mutation frequencies. We quantified the background mutation frequencies of amino acid mutations across commonly mutated tumor suppressors and oncogenes with dinucleotide-based mutation rates[39]. In brief, dinucleotide-based mutation rates were derived from a nonreversible mutation model based on alignments between human and mouse non-coding DNA sequences from human chromosomes 10 and 21. In this mutational model, there are no external mutational pressures such as those derived from UV radiation or toxins such as aflatoxin. Since we observed such strong mutation distribution conservation across databases, we posited that although such mutational signatures may be relevant, intrinsic mutational processes should be the main determinant of background mutation rates. The internal CpG-associated C $\rightarrow$ T mutation signature is an order of magnitude more common than the other types of mutations. For each nucleotide position across each gene, we assign a mutational rate which is the average of the left and and right neighboring dinucleotide mutation rates for dinucleotides containing the position in question, effectively assigning a trinucleotide mutation rate for single nucleotide mutations. To derive the background mutation frequencies, we first assign each nucleotide in the coding region of each gene an effective trinucleotide mutation rate. Next, for each amino acid mutation we sum the mutation rates of all of its possible nucleotide mutations. Finally, we normalize the rates to create a probability distribution.

Let l_n and r_n be the rates of mutation of a nucleotide n corresponding to its left and and right dinucleotides. Further, let the set N_m correspond to all of the nucleotide mutations that result in amino acid mutation m. We set the average rate of mutation for a nucleotide n as $\mu_n = (l_n + r_n)/2$. We set the background mutation frequency of an amino acid mutation by normalizing across all amino acid mutation rates,

$$p_m = \frac{\sum\limits_{n \in N_m} \mu_n}{\sum\limits_{m'} \sum\limits_{n' \in N_{m'}} \mu_{n'}} \,. \tag{1}$$

We consider the full gene sequence with both introns and exons, which we downloaded from the National Center for Biotechnology Information (NCBI)[40]. All nucleotides within the coding region of each gene have a right and left neighboring nucleotide, i.e. there are no boundary cases. The amino acid alterations corresponding to each nucleotide mutation were determined with the SnpEff software package (Version 4.3)[41].

Definition of conservation and neoantigen presentation

Amino acid conservation. We defined the conservation of a driver gene mutant as the conservation of the wild-type amino acid with homologs to the driver gene protein. Amino acids which are conserved may play a role in function and protein structure, as well as protein-protein binding. Mutations in conserved amino acids may contribute to the cancer phenotype more so than not-conserved amino acids.

In brief, we infer conservation via evolutionary rates for each amino acid across commonly mutated tumor suppressors and oncogenes in cancer similar to the default parameters of the ConSurf server[42,43]. For each protein sequence (except p53), we find homologous sequences available within the Uniref90 database[44,45] using the `phmmer` software (`HMMER` Version 3.3.2, `hmmer.org`) with an E-value cutoff of 0.0001. We then select 150 equally-spaced homologous Uniref90 protein sequences. Next, we cluster the sequences using `cdhit`[46,47] with a sequence identity threshold of 0.95. We then align the 150 homologous sequences with the original protein sequence using the `MAFFT` software (Version 7.475[48]). Finally, we run the `rate4site` software (Version 3.0.0[49]) to assign a standardized evolutionary rate to each amino acid of the protein sequence. For p53, we used 33 pre-determined homologous protein sequences across species used in previous work[3] using the ConSurf server with default parameters. The evolutionary rates are defined so that the average of them is zero and the standard deviation is one. Lower evolutionary rates indicate increased conservation.

We determined the degree to which hotspots were in conserved amino acids, with respect to the other non-hotspot mutations within the same gene, using the two-sided Welch's T-test.

Neoantigen presentation. We define the neoantigen presentation of a driver gene mutant as a function of the neoepitopes derived from the mutation and the germline MHC-I haplotype in the person with the cancer. We infer dissociation constants $K_I^m(p,h)$ in nM units from computationally-derived IC50 values calculated from NetMHC 3.4 and NetMHC 4.0[50–53].We estimated the effective mutant peptide MHC-I affinities for all missense mutants by computing:

$$\frac{1}{{K_{\text{eff}}}_I^m} = \left\langle \max_{\substack{p\in P\\ h\in H}} \frac{1}{K_I^m(p,h)} \right\rangle_{H\in D_H}, \tag{2}$$

where p is a peptide, P_m is the set of mutated peptides around mutation m, h is an HLA-I within the set H of germline HLA-I, $K_I^m(p,h)$ is the predicted dissociation constant between a mutant peptide and an HLA-I molecule, and D_H is a population of MHC-I haplotypes. Here we consider all mutated peptides of length nine, the most common length presented[51]. Typically, $|P_m| = 9$ and $|H| = 6$; exceptions may occur if the mutation occurs close to the edges of the protein or if there are additional mutations which reduce MHC-I expression. The peptides are derived from canonical protein transcripts as determined by UniProt[44]. The "reduced neoantigen presentation" is computed as a two-sided Welch's T-test between the hotspot mutations' versus the non-hotspot mutations' effective mutant peptide MHC-I affinities (Eq. 2) for a particular driver gene.

Inference of apparent dimer dissociation constants

In order to estimate the functional capacity of all possible p53 missense mutants, we leveraged a transactivation yeast assay dataset[5]. In that work, all possible p53 missense mutations derived from single nucleotide mutations were mono-alleleically expressed in yeast cells in which eight target promoter sequences were tagged with either enhanced green fluorescent protein (EGFP) on the p21*WAF1* target sequence or Ds-Red on the other seven target genes (*MDM2*, *BAX*, *GADD45*, *h1433s*, *p53AIP1*, *NOXA*, and *p53R2*). The p21*WAF1* and *MDM2* sequences are human-derived, and the others were synthetic with p53 response elements. Fluorescence intensity was measured for each mutant. The average relative fluorescence intensity of each p53 mutant was reported with respect to wild-type p53.

Under such conditions, it is assumed that all of the mutant and wild-type p53 proteins are expressed at equally-low concentration. Therefore, we expect the relative transactivation values reported are largely driven by the different affinities of each mutant to the target DNA sequence. p53 monomers have a tendency to oligomerize as dimers or tetramers[23]. p53 primarily transactivates target DNA in a highly-cooperative manner as a tetramer, i.e. a “dimer of dimers”[54]. which may sequentially bind the same promoter sequence. The affinity of the second dimer is typically much larger than the affinity of the first dimer due to cooperativity. The “effective dimer dissociation constant” is equal to the geometric mean of the two dimer dissociation constants. The effective dimer dissociation constants of truncated wild-type p53 (DNA-binding domain and oligomerization domain, amino acids 94-360) to well-known targets of p53 transactivation have also been quantified *in vitro*[55]. For promoter sites with multiple binding sites, we take the geometric mean of the affinities as the effective affinity. Additionally, it has been shown that the N- and C- termini of p53 regulate DNA binding, as they non-specifically bind to DNA and reduce the effective affinity of the dimer complex to a specific sequence, with an approximately 10-fold reduction in specific binding affinity both *in vitro* and *in vivo*[56–58]. Furthermore, the termini contain residues that are targets of post-translational modification such as acetylation[59] which may or may not be post-translationally modified. Therefore, we correct for the full-sequence dissociation constant by multiplying the reported dissociation constants by a factor of 10 in order to correct for the termini.

The likelihood of p53 binding a target sequence will involve both the p53 concentration and the amino acid sequence-based binding affinity. We interpret the probability that p53 binds to a target DNA promoter sequence via a Hill function with cooperativity of two[54]:

$$P^m_{\text{DNA}} = \frac{(L_{\text{REF}})^2}{(L_{\text{REF}})^2 + (K^m_{\text{DNA}})^2}\ , \tag{3}$$

where P^m_{DNA} is the probability of a mutant m binding to target DNA, L_{REF} is the concentration of p53 dimer in the yeast assay, and K^m_{DNA} is the effective dimer dissociation constant of binding a DNA promoter sequence for mutant m.

For each missense mutation, the yeast assays report an averaged relative transactivation value for each target promoter sequence, which we define as:

$$T^m_g = \frac{F^m_g}{F^{\text{wt}}_g}\ , \tag{4}$$

where T^m_g is the ratio of the mutant fluorescence F^m_g over the wild-type fluorescence F^{wt}_g for a particular target gene promoter sequence g and mutant m.

We assume the fluorescence value is in the linear range of the binding curve, so that the fluorescence of wild-type or mutant p53 binding DNA is equally proportional to its probability of binding DNA. The relative transactivation value can then be estimated as:

$$T_g^m = \frac{(L_{\mathsf{REF}})^2 + (K_{T_g}^{\mathsf{wt}})^2}{(L_{\mathsf{REF}})^2 + (K_{T_g}^m)^2} \,, \tag{5}$$

where $K_{T_g}^{\mathsf{wt}}$ and $K_{T_g}^m$ are the effective dimer dissociation constants for the wild-type homotetramer and the mutant homotetramer and a specific DNA target sequence g, respectively. Therefore, we can transform the mutant-specific relative transactivation T_g^m to the mutant-specific dissociation constant $K_{T_g}^m$ via:

$$K_{T_g}^m = \sqrt{\frac{(K_{T_g}^{\mathsf{wt}})^2 + (L_{\mathsf{REF}})^2(1 - T_g^m)}{T_g^m}} \simeq \frac{K_{T_g}^{\mathsf{wt}}}{\sqrt{T_g^m}} \,, \tag{6}$$

where the final approximation arises since the dissociation constants tend to be of order $\geq 10^2$ nM, as the ratio of flourescence values is bounded in the experimental data by $0 \lesssim T_g^m \lesssim 4.6$. We choose a reference concentration for a p53 dimer, L_{REF}, of approximately 1 nM that is consistent with a previously-defined low-concentration regime of p53 in yeast[60]. To account for non-specific binding, we add an offset of 9×10^{-4}, which is an order of magnitude lower than the lowest non-zero transactivation value in the experiment ($T_g^m = 0.001$).

Note there is a non-linear relationship between the two variables, with lower T_g^m values corresponding to higher $K_{T_g}^m$ values. We define the functional category of p53 mutants using the IARC definitions of T_g[61].

The transcriptional activity T_A^m of a *TP53* mutant is defined as the median of the association constants derived from Eq. 6 across genetic targets:

$$T_A^m = \underset{g \in G}{\mathsf{med}} \left[\frac{1}{K_{T_g}^m} \right] \,. \tag{7}$$

Inference of tissue- and mutant-specific concentrations

Under normal conditions, wild-type p53 is maintained at low concentrations and has a half-life of approximately 20 minutes largely via a negative-feedback loop with MDM2[22]. Under conditions of stress, wild-type concentration typically rises, increasing transactivation of target genes responsible for cellular stress response. Missense-derived mutant p53 concentration tends to increase to non-typically high levels that are both tissue- and mutant-specific, while nonsense mutations tend to strongly reduce p53 concentration[12-14].

The fitness model strongly depends on the mutant concentration, as it links both the functional and immune components via a biophysical binding model. However, quantitative concentration information for most p53 mutants is unavailable. To address this, we aimed to infer each missense mutant's concentration. p53 concentration is directly regulated by MDM2 and p53 mutants alter the ability for the transcription factor to bind promoter sites on DNA, such as the *MDM2* promoter site. From this, we expect that mutants which retain *MDM2* promoter DNA capacity will induce wild-type p53 comparable levels of MDM2, which will in turn constrain p53 concentration to wild-type levels. Mutants which greatly reduce p53 binding of *MDM2* promoter DNA will reduce the amount of circulating MDM2, thus permitting a higher concentration of mutant p53. We leverage this principle and apply it to a TCGA proteomics dataset to infer tissue- and mutant-specific concentrations utilizing inferred mutant DNA-binding affinities from previous work in yeast[5]. In doing so we quantify the role of the p53-MDM2 negative feedback loop in a large dataset such as TCGA. We describe the methods in detail below.

Quantifying mutant p53 concentrations. For a particular sample, the concentration of p53 will depend on the tissue type. It will also depend on the mutational status and the number of mutant/wild-type p53 alleles available. It will also strongly depend on tumor heterogeneity, such as the clonal status of the mutation and the purity of the sample. We aimed to quantify the distribution of mutant p53 concentration using TCGA, where the Reverse-Phase Protein Assay (RPPA) has been used to quantify relative protein expression in TCGA samples. We downloaded Level 4 RPPA data (TCGA-PANCAN32-L4) from The Cancer Proteome Atlas (TCPA)[62,63]. In a manner similar to a Western blot, protein expression is inferred via fluorescence from a tagged antibody. To account for batch effects, the inferred $\log_2$ concentration values are median-normalized by subtracting each sample's value with two medians – the median $\log_2$ concentration for that protein across all samples and the median $\log_2$ concentration of all of the proteins in one sample. The value reported is proportional to the $\log_2$ of the true concentration in the sample. If we define RPPA reported values as R, the subtracted constants as c and assume that c is distributed around a central value, and the protein concentration as L, then $R = \log_2 L - c$ or $L = C \times 2^R$, where C is a constant which provides the appropriate units. We show the values for C, and by extension, c, are distributed around a central value for wild-type p53.

Multiple efforts have tried to quantify wild-type p53 concentration in cells under different conditions[22,23], typically converging on concentrations on the order of 10^2 to 10^3 nM across different cell types. In MCF-7 wild-type p53 breast cancer cell lines, the average concentration is estimated at approximately 150 nM[22]. We leverage this value to define the constant C, with appropriate nanomolar units (nM) using TCGA wild-type breast cancer (BRCA) p53 RPPA data. An average concentration of 150 nM of p53 in MCF-7 breast cancer cells with two wild-type p53 alleles means that we expect each p53 allele to contribute approximately 75 nM. In order to find the equivalent protein expression in the RPPA dataset, we examined the distribution of 2^R per allele in p53 wild-type breast cancer cells (BRCA) in TCGA. We selected samples for which there were no p53 mutations nor amplifications/deletions. In general, for each p53-mutated TCGA sample, when possible, we: (1) estimate the purity of the sample, (2) estimate the

clonal status of the p53 mutation in the tumor, (3) infer the number of p53 alleles, and (4) distinguish which p53 alleles are wild-type and mutant. This methodology is described below.

Estimating tumor heterogeneity. The p53 RPPA value for a mutant p53 tumor sample in TCGA is not entirely due to the mutated p53. The p53 RPPA value of a tumor sample may be decomposed as:

$$2^{R_S} = 2^{R_{\text{wt}}}[C_N(1-p) + C_T p(1-f) + (C_T - N_m)pf] + 2^{R_m}[N_m pf] \,, \quad (8)$$

where R_S is the sample p53 RPPA value, R_{wt} is the wild-type p53 component of the sample RPPA, R_M is the mutant p53 component of the sample RPPA, C_N is the expected p53 ploidy in typical, non-cancerous cells, p is the purity of the sample, f is the cancer cell fraction, C_T is the number of p53 alleles in tumor cells, and N_m is the number of mutant alleles in a p53-mutant cell. The components may be justified as follows:

- WT p53 alleles from the normal portion of the sample: $C_N(1-p)$
- WT p53 alleles from the tumor portion of the sample without p53 mutations: $C_T p(1-f)$
- WT p53 alleles from the tumor portion of the sample with p53 mutations: $(C_T - N_m)pf$
- MT p53 alleles from the tumor portion of sample with p53 mutations: $N_m pf$

subject to the following constraints: $C_N = 2$ (typical p53 ploidy), $C_T \geq 0$, $N_m \geq 0$, $0 \leq N_m \leq C_T$, $0 \leq p \leq 1$, and $0 \leq f \leq 1$. For example, if $C_T = 2$ and $N_m = 1$, then the cell is heterozygous in mutant p53, and if $N_m = 2$, then it is homozygous in mutant p53.

The purity of TCGA samples was quantified with ASCAT[64] and downloaded from COSMIC[65]. Copy number variation data for TCGA samples was downloaded from the National Cancer Institute's Genomic Data Commons repository[66]. For processing of p53 copy number variation data, we averaged all p53 copy number values, after converting from segmentation values ($C_T = 2 \times 2^{\text{seg}}$, where seg is the segmentation value), overlapping with the *TP53* gene region (defined as chr17 : [7661779, 7687550], reference genome GRCh38). Neither the cancer cell fraction nor the zygosity of p53 mutants in TCGA have been previously quantified, which we compute in the next section. Knowing these quantities allows us to solve for 2^{R_M}, a value that is proportional to the concentration of one mutant p53 allele.

Estimating cancer cell fraction f and the number of mutant alleles N_m. Sequencing of DNA from tumor samples provides the number of reads that cover a mutation. It indicates the number of reference and alternate alleles, given a reference genome. If we define the number of reference allele reads as R_r, the number of alternate allele reads to R_a, and the variant allele fraction as V, we have:

$$V = \frac{R_a}{R_r + R_a} \,, \quad (9)$$

where $0 \leq V \leq 1$. The variant allele fractions for p53 mutations in TCGA were downloaded from the Genomic Data Commons repository,[66] the values of which were averaged across mutation callers. Theoretically, the variant allele frequency can also be defined as[67–70]:

$$V = \frac{pfN_m}{C_N(1-p) + C_T p} \,. \quad (10)$$

We have estimates for all variables except f and N_M. The term $w = fN_M$ is defined as the multiplicity. The probability distribution of the number of reads that align to a mutation may be interpreted in terms

of a binomial distribution, where R_a is the number of successes and $R_r + R_a$ is the number of trials. We find the value of w that maximizes the posterior distribution. We treat f and N_m as independent. We calculate f by computing the value that maximizes the likelihood of getting a sample variant allele frequency according to the following procedure:

1. For each sample in TCGA we obtain a value of R_r, R_a, and V for a p53 mutant.
2. We vary f from 0.01 to 1 for 100 evenly-spaced values and calculate V across the variations of f.
3. We calculate the probability of getting R_a successes given $R_r + R_a$ trials given a probability V for each varied f. $p = B(R_r + R_a, V) = \binom{R_r+R_a}{R_a} V^{R_a}(1-V)^{R_r}$.
4. We then normalize the probability distribution and find the cancer cell fraction that maximizes the binomial probability. This is f_{opt}.
5. Finally, we solve for N_m using the actual V from TCGA sample and round it to an integer. If $N_m > C_T$, then we set $N_m = C_T$.

Having these components for TCGA tumors, we can estimate the heterozygosity of p53 mutations, the concentration of the mutant alleles in a sample, and the typical concentrations of different p53 mutants. Furthermore, we can quantify the MDM2 and p53 negative-feedback loop from such data as a check for consistency. There is no RPPA information available for the MDM2 protein, but there is RNA expression data available. As MDM2 is transactivated by p53, we expect MDM2 RNA expression (quantified in Transcripts Per Million (TPM)) to be proportional to MDM2 concentration, and negatively related to p53 concentration. Similarly, we expect the p53 concentration to be positively correlated with p53 RNA expression as a check on self-consistency.

Computing the effective p53 MDM2 promoter affinity in TCGA samples. By normalizing by the number of mutant p53 alleles, the methods outlined above allow us to infer the per-allele mutant concentration in a particular sample. Next, we predicted the level of MDM2 transactivation within a cancer cell based on the estimated distribution of mutant and wild-type p53 alleles. Samples in TCGA may contain different distributions of the number of MT and WT alleles, as some may be heterozygous in a p53 mutation, others may be homozygous, and others may have deletions/amplifications in the *TP53* gene. A sample with both wild-type and mutant *TP53* alleles will not only contain fully mutant and fully wild-type tetramers, but to a larger extent will also contain a distribution of hybrid wild-type and mutant tetramers. Previous work has attempted to quantify the effect that mixed mutant and wild-type tetramers have on binding affinity, suggesting that hybrid wild-type and mutant p53 tetramers are not fully inactivated, taking approximately three mutant p53 monomer subunits to truly render a p53 tetramer non-functional for certain mutations[71].

The dissociation constant used in the cooperative Hill function for the functional term is the apparent dimer dissociation constant, defined as the geometric mean of the sequential dissociation constants of two dimers to same promoter region, where the first is large and the second is small[54]. We can infer the wild-type dimer dissociation constant from previous work[55], and we earlier estimated the dimer dissociation constant for a fully-mutant p53 tetramer. In order to estimate the effective dissociation constants associated with mixed wild-type/mutant p53 tetramers, we assume that an equally-mixed tetramer composed of one WT:WT dimer and one MT:MT dimer must have the same binding efficiency as one composed of two WT:MT dimers. The dimer dissociation constant of an equally-mixed tetramer is assumed to be $K_T^{\text{MT,WT}} = \sqrt{K_T^{\text{2WT}} \times K_T^{\text{2MT}}}$. By similar logic, the dimer dissociation constant of a 3 WT

: 1 MT mixed tetramer is assumed to be $\sqrt{K_T^{\text{2WT}} \times K_T^{\text{MT,WT}}}$, and the dimer dissociation constant of a 1 WT : 3 MT mixed tetramer is assumed to be $\sqrt{K_T^{\text{2MT}} \times K_T^{\text{MT,WT}}}$.

The probability of a particular tetramer species existing will depend on the number of WT and MT *TP53* alleles in a sample. If we define the total number of *TP53* alleles as $N_{\text{Tot}} = C_N + C_T$, the number of *TP53* wild-type alleles as C_N, and the number of *TP53* mutant alleles as C_T, then the probability of a wild-type monomer incorporated into a tetramer is $q_w = C_N/N_{\text{Tot}}$, and the corresponding mutant probability is $q_m = C_T/N_{\text{Tot}}$.

The probability of a tetramer λ is then:

$$P_\lambda = P(X, Y = 4 - X) = \binom{N_{\text{Tot}}}{X} q_w^X q_m^Y \,, \tag{11}$$

where X is the number of wild-type monomer units in the tetramer, Y is the number of mutant monomer units in the tetramer, and $X + Y = 4$ for all tetramers $\lambda \in \Lambda$.

We define the effective association constant of MDM2 promoter as the expectation value across tetramer species, weighted by their probability of being formed in a cell:

$$\left\langle \frac{1}{K_T} \right\rangle_\Lambda = \sum_{\lambda \in \Lambda} P_\lambda \frac{1}{K_T^\lambda} = \binom{N_{\text{Tot}}}{X} q_w^X q_m^Y \frac{1}{K_T^\lambda} \,. \tag{12}$$

Now we can determine if there are any relationships between the effective MDM2 promoter association constant and the per-allele corrected concentration across TCGA samples with available data. In pan-cancer and tissue-specific settings, we plot all of the unique *MDM2* promoter association constants versus the median of the per-allele concentrations corresponding to that association constant to control for noise. We fit a line to the data using a least-squares regression, which defines a quantitative expression for the relationship between normalized mutant p53 concentration and expected MDM2 transactivation by p53 across missense mutants. The predominantly negative relationships between the expected *MDM2* association constant and the p53 concentration provide additional evidence for the p53-MDM2 negative feedback loop in TCGA and allow us to estimate tissue- and mutant-specific concentrations based on the regression line for mutations with unavailable concentration data.

In all cases, the relationship between the effective *MDM2* promoter association constant and p53 concentration is given by the expression:

$$\log_2 L_p^m = a \times \left\langle \frac{1}{K_T} \right\rangle_\Lambda + b \,, \tag{13}$$

where $\log_2 L_p^m$ is the $\log_2$ of the per-allele concentration of mutant p53 monomers, $\langle \frac{1}{K_T} \rangle_\Lambda$ is the effective association constant of MDM2 promoter across the Λ tetramers, and a and b are the slope and intercept that are being fit. We present fitness models in both the pan-cancer setting and a tissue specific model for colorectal cancer. In the pan-cancer setting, $a = -133.06$ and $b = 8.68$.

Free fitness model

Fitness model components. We propose a minimal biophysical model of the fitness advantage a tumour acquires from a *TP53* mutation in order to explain the observed population mutation frequency distribution. We expect higher fitness *TP53* mutations are more likely to be fixed in tumors and therefore will have a higher observed mutation frequency, and the opposite will occur for less fit mutations.

The relative fitness of a mutation m for a patient with HLA haplotypes $H = [A_1, A_2, B_1, B_2, C_1, C_2]$ is defined by the following fitness function:

$$f_m(H) = \sigma_T T_m + \sigma_I I_m(H) \equiv f_m^T + f_m^I(H)\,, \tag{14}$$

where the term $\sigma_T T_m$ defines the "functional fitness", f_m^T (the effect a *TP53* mutation has on mutant p53 transcription factor-associated binding activity), and $\sigma_I I_m(H)$ defines the "immune fitness", $f_m^I(H)$ (corresponding to the immunogenicity of the mutant peptides generated by a *TP53* mutation, which depends on the set of HLA-I molecules in haplotype H). The parameters $\{\sigma_T, \sigma_I\}$ assign relative weights to the fitness components and set the overall scale of the fitness amplitude. They are optimized to fit the training set in our model.

We define T_m as the median probability that a mutant p53 homotetramer does not bind target promoter sites in DNA across the eight target genes (*WAF1*, *MDM2*, *BAX*, *h1433s*, *AIP1*, *GADD45*, *NOXA*, and *P53R2*) for which we have data available from previous work defining mutant p53 binding in a quantitative yeast assay[5]. T_m is modeled by a cooperative Hill function with a cooperativity coefficient of two[54],

$$T_m = \frac{(K_T^m)^2}{(L_D^m)^2 + (K_T^m)^2}\,, \tag{15}$$

where L_D^m is the concentration of a mutant p53 homodimer for mutation m, which is equivalent to half of the total mutant p53 monomer concentration, and K_T^m is the median apparent dimer dissociation constant for binding target DNA across the eight target genes studied for mutation m. The methods for computation of K_T^m and L_D^m are described in detail in the subsequent sections.

We define $I_m(H)$ as the geometric mean of the predicted probabilities of all mutant peptides binding class-I MHC molecules for mutation m, via a non-cooperative Hill function,

$$I_m(H) = \left(\prod_{p \in P_m,\, h \in H} \frac{L_p^m}{L_p^m + K_I^m(p,h)} \right)^{\frac{1}{|P_m| \times |H|}}\,, \tag{16}$$

where p is a peptide, P_m is the set of mutated peptides around mutation m, h is an HLA-I within the set H of germline HLA-I, L_p^m is the concentration of the peptide (which is also the p53 monomer concentration and twice the p53 dimer concentration), and $K_I^m(p,h)$ is the predicted dissociation constant between a mutant peptide and an HLA-I molecule.

We infer the concentrations L_p^m and $L_D^m = L_p^m/2$ from TCGA in nanomolar (nM) units. We also consider alternative fitness models with additional components, which we discuss in the section on model performance and comparison.

Let mutation m occur in a patient with MHC-I haplotype H. The relative contribution of a mutation to the growth of the tumor clone with this mutation is described by exp $[(f_{\text{wt}} + f_m(H;\sigma_T,\sigma_I))]$, where f_{wt} is the background growth rate of the tumor clone without the mutation and f_m is the fitness effect

of mutation m. Given all possible mutations and their background frequencies, p_m, as determined by the background mutation rates, the model-predicted frequency of an observed mutation m within the haplotype H is given by

$$\hat{x}_m(H) = Z_H^{-1} p_m \exp\left[f_m(H)\right] = Z_H^{-1} p_m \exp\left[f_m^T + f_m^I(H)\right] \,, \tag{17}$$

where $Z_H = \sum_m p_m \exp\left[f_m(H)\right]$. Since we consider the relative frequencies of mutations, the constant wildtype growth term f_{wt} factors out from the above expression. To incorporate the effect of background mutations on equal footing with the fitness terms we can define the "free fitness" of a mutation, F_m, as

$$F_m \equiv \log(p_m) + f_m^T + f_m^I(H). \tag{18}$$

F_m, now a free fitness function, serves an analogous purpose to a negative free energy in statistical physics. The free fitness of a mutation may be conveniently represented as a point, P_m, in a phenotypic space:

$$P_m = (\log(p_m) + f_m^T, f_m^I(H)). \tag{19}$$

Such a representation of the free fitness landscape therefore serves as a genotype-to-phenotype mapping. In the text we refer to $\log(p_m) + f_m^T$ as an "intrinsic fitness", since it refers to processes intrinsic to the cancer cell, and $f_m^I(H)$ as an "extrinsic fitness", since it refers to effects on the cell from its environment.

The population level predictions for frequencies of mutations are computed as the expectation value over the database of haplotypes D_H representative of a population,

$$\hat{x}_m = \langle \hat{x}_m(H) \rangle_{H \in D_H} \,. \tag{20}$$

The mutation frequency predictions depend on the fitness model parameters: $\hat{x}_m \equiv \hat{x}_m(\sigma_T, \sigma_I)$. Each mutation occurs within a *TP53* codon. We define the codon mutation frequency as the sum of the missense mutation frequencies that alter a codon's amino acid (i.e. all missense mutations within a codon). For instance, the codon frequency at position R175 is the sum of all individual missense mutations which alter the arginine corresponding to codon 175. This step is done as an additional check on the predictive power of the fitness model, as the p53 mutation hotspots are clustered in a set of well-defined hotspot codons.

The relative fitness of a *TP53* mutation defines whether or not its population frequency increases or decreases with respect to the background mutation frequency. Higher fitness mutations will increase their population frequency with respect to their background mutation frequency, and lower fitness mutants will have a lower population frequency with respect to their background mutation frequency. We define the predicted ratio $\hat{W}_m = \hat{x}_m / p_m$ as the relative increase or decrease of the predicted frequency with respect to the background mutation frequency for mutation m, and the posterior ratio $W_m = x_m / p_m$ as the relative increase or decrease of the observed mutation frequency with respect to the background mutation frequency for mutation m. These terms are the Wrightian fitness of a mutation – ratios which are > 1 indicate population frequency growth, and ratios which are < 1 indicate population frequency decrease.

Haplotype distributions. We train the weights for our model on the missense p53 mutation frequencies and haplotypes available within TCGA[72]. Our training cohort was chosen since these are non-simulated haplotypes with full MHC-I linkage information. There are a total of 8,507 haplotypes which correspond to 6,379 unique haplotypes. For testing the relevance of the sampled haplotype space on the

fitness model predictions, we used marginal haplotype frequencies from the National Marrow Donor Program (NMDP) database corresponding to European Caucasian-Americans, which provides information on 1,242,890 donors[73,74]. Within this database, there is no extensive haplotype information but there is extensive individual HLA population frequency information.

We assume the MHC-I haplotype will consist of two each of HLA-A, HLA-B, and HLA-C for a total of 6 MHC-I genes. We assume a multinomial distribution with an independent frequency model without MHC-I linkage for each HLA-A, HLA-B, and HLA-C gene. We constructed all possible haplotypes using all available MHC-I within the database. The number of heterozygous HLA-I genes is given by N_H, where $N_H \in [0, 1, 2, 3]$. The probability of a haplotype $H = [A_1, A_2, B_1, B_2, C_1, C_2]$ is given by:

$$p(H) = 2^{N_H} \prod_{h \in H} p_h \,, \tag{21}$$

where p_h corresponds to the marginal probability of HLA-I h within haplotype H.

We sort the haplotype probabilities and take a subset of the most frequent haplotypes. We compute the expected mutation frequency for each haplotype and calculate a weighted average across the population, with weights given by the expected haplotype probabilities, resulting in the expected mutation population frequency according to Eq. 20.

Pareto optimality. We compute the Pareto front for our data as follows: we query each mutation m and its corresponding point P_m in phenotypic space and compare it to every other mutation n and its corresponding phenotype point P_n. A mutation not on the Pareto front is one for which there exists a point in phenotypic space for which one feature is improved while the other is at least equal.

Specifically, for each pair of mutations m and n we consider the two differences between their coordinates in the phenotypic space:

$$(\log p_m + f_m^T) - (\log p_n + f_n^T) = \log\left(\frac{p_m}{p_n}\right) + (f_m^T - f_n^T) = d_1 \,, \tag{22}$$

and

$$f_m^I - f_n^I = d_2 \,. \tag{23}$$

For a mutation m, if d_1 or d_2 are greater or equal to $\epsilon = 0.1$ for all other mutations in the phenotypic space, then point P_m is on the Pareto front. To illustrate the Pareto front, we draw a convex hull containing the Pareto front coordinate set using the *shapely* Python package smoothed using the following parameters: `pareto_front.buffer(10, join_style=1, mitre_limit=50).buffer(-10, join_style=1, mitre_limit=50)`.

We then truncate the the convex hull based on the maximum of the intrinsic fitness, $\log p_{m_i} + f_{m_i}^T$, and the maximum of the immune fitness, $f_{m_i}^I$, and do not close the convex hull, allowing the Pareto front to be delimited by the Pareto optimal coordinate set. To obtain the optimal solution on the Pareto front, we calculate the point on the Pareto front with the maximum free fitness by discretizing the Pareto front into 10,000 equally spaced points and calculating the free fitness value for each point.

Model training

Model fitting. To optimize the fitness model parameters, $\Theta = \{\sigma_T, \sigma_I\}$, we minimize the cross entropy between the observed mutation frequencies x_m and the frequencies predicted by the model, $\hat{x}_m$,

$$\mathcal{H}(x_m, \hat{x}_m, ; \Theta) = -\sum_m x_m \log \hat{x}_m(\Theta) \; . \tag{24}$$

Minimization of the cross-entropy is equivalent to the minimization of the Kullback-Leibler divergence between the distributions of the observed and predicted frequencies and to maximization of the likelihood of the mutation data under the given fitness model. Each unique observed mutation m in the database D_m is predicted to occur with probability $\hat{x}_m(\Theta)$. The data log-likelihood under our model is given by:

$$\mathcal{L}(D_m|\Theta) = \sum_{m \in D_m} \log \hat{x}_m(\Theta) = -n\mathcal{H}(\Theta) \; , \tag{25}$$

where $n = |D_m|$ is the size of the database of p53 mutations. We minimize the cross entropy using the limited-memory Broyden–Fletcher–Goldfarb–Shanno algorithm (L-BFGS) with the analytically-computed gradient of the cross-entropy. We find that the optimized parameters for pan-cancer TCGA are $\sigma_T^* = 4.152$ and $\sigma_I^* = -42.212$.

Alternative models. We compared our minimal model in Eq. 14 to alternative models of varying complexity. To assess the predictive power of individual components, we performed model decompositions, where only a subset of components was used. We also examined more complex models which include other phenotypes of p53.

Models without selection. These models account for the mutation rates only and assume no selection on the mutations ($f_m = 0$). We consider a uniform model and a model of dinucleotide-specific frequencies. The predicted frequencies of mutations will therefore reflect the background distributions:

$$\hat{x}_m = 1/N \; , \tag{26}$$
$$\hat{x}_m = p_m \; . \tag{27}$$

Partial models. In these models the background distribution of mutations is assumed to follow the dinucleotide-based estimation. We consider decompositions of the minimal fitness model into individual components:

$$f_m = \sigma_T T_m \; , \tag{28}$$
$$f_m(H) = \sigma_I I_m(H) \; . \tag{29}$$

Extended functional models. For extended models, we included all target genes in set G (*WAF1*, *MDM2*, *BAX*, *h1433s*, *AIP1*, *GADD45*, *NOXA*, and *P53R2*) for evaluating the transactivation component of the fitness model:

$$f_m = \sum_{g \in G} \sigma_T^g {T^g}_m \; , \tag{30}$$
$$f_m(H) = \sum_{g \in G} \sigma_T^g {T^g}_m + \sigma_I I_m(H) \; . \tag{31}$$

For each additional model, we retrained the parameters using COSMIC- and TCGA-derived mutations as well as TCGA-derived haplotypes.

Protein conservation and stability.

We also evaluated an additional stability term for each p53 mutant. Previous work has suggested different mutants have different temperature-dependent stabilities that do not necessarily reflect functional binding of target DNA[75]. The stability of the protein is quantified as the change in free energy between the natural and denatured state. If the change in free energy of folding is zero, then at equilibrium there will be an equal amount of folded and unfolded protein, as both the unfolded and folded states are equally likely. The fraction of stable folded protein, which is equivalent to the probability that the protein is folded properly (defined as S_m) is defined as:

$$S_m = \frac{1}{1 + \text{exp}^{-\Delta G/RT}} , \tag{32}$$

where R is the gas constant, T is the temperature in Kelvin, and ΔG is the change in free energy.

Previous work experimentally quantified ΔG for a number of mutants[75]. Structural algorithms have been developed that are highly correlated to the experimental values previously reported[76]. In order to quantify the changes in free energy for all possible mutants, we use values from the PBSA algorithm, as it had the highest linear correlation to experimental values[76]. The PBSA algorithm only reports on the DNA-binding domain of p53 (here defined as amino acids between 96 to 289) as available crystal structures for p53 largely report on this region. For the other mutants outside of the defined DNA-binding domain, we assume they do not alter protein stability, and are assigned $\Delta G = 0$, which is consistent with the fact that these regions are largely disordered and difficult to structurally characterize[77].

The PBSA algorithm reports $\Delta\Delta G$, which is defined as[76]:

$$\Delta\Delta G = \Delta G^{M}_{\text{N}-\text{D}} - \Delta G^{W}_{\text{N}-\text{D}} , \tag{33}$$

where $\Delta G^{M}_{\text{N}-\text{D}}$ is the change in free energy for the mutant, and $\Delta G^{W}_{\text{N}-\text{D}}$ is the change in free energy for the wild-type, both for the natured-to-denatured direction. The value of $\Delta G^{W}_{\text{N}-\text{D}}$ has been reported by extrapolation to be approximately -3 kcal/mol[75]. We then solve for $\Delta G^{M}_{\text{N}-\text{D}}$ for the mutant change in free energy across mutants.

Models across commonly mutated tumor suppressors and oncogenes. For all considered commonly mutated tumor suppressors and oncogenes, we considered models of varying complexity. We considered models with only a dinucleotide background, as in Eq. 27. Additionally, defining the conservation term as $\sigma_C C_m$, we also considered models with only conservation predictions over a dinucleotide background,

$$f_m = \sigma_C C_m , \tag{34}$$

only *in silico* immunogenicity predictions (as in Eq. 2) over a dinucleotide background,

$$f_m = \sigma_I \frac{1}{{K_{\text{eff}}}_I^m} , \tag{35}$$

and combined models with both conservation and immunogenicity components over a dinucleotide background,

$$f_m = \sigma_C C_m + \sigma_I \frac{1}{{K_{\text{eff}}}_I^m} \ , \tag{36}$$

For *KRAS*, there is additional function information available for seven hotspot mutants (G12A/C/D/R/V, G13D, and Q61L)[25]. We included the additional functional information for just these mutants as an additional level of complexity for modeling KRAS mutations,

$$f_m = \sigma_C C_m + \sum_{q \in Q} \sigma_F^q {F^q}_m + \sigma_I \frac{1}{{K_{\text{eff}}}_I^m} \ , \tag{37}$$

where the term $\sum_{q \in Q} \sigma_F^q {F^q}_m$ considers functional phenotypes F^q within the set of Q functional phenotypes, which are intrinsic and extrinsically-assisted GTPase activity, as well as downstream binding to RAF effector protein. Notably, of these functional phenotypes for *KRAS* only the downstream binding to RAF effector was predictive of the *KRAS* mutation frequencies.

The structural term $\sigma_S S_m$ is only available for p53. For p53, we consider fitness models that are extended by the protein conservation and stability terms, across two versions of the functional component, namely:

$$f_m(H) = \sigma_T T_m + \sigma_I I_m(H) + \sigma_C C_m \ , \tag{38}$$

$$f_m(H) = \sum_{g \in G} \sigma_T^g {T^g}_m + \sigma_I I_m(H) + \sigma_C C_m \ , \tag{39}$$

for conservation and

$$f_m(H) = \sigma_T T_m + \sigma_I I_m(H) + \sigma_S S_m \ , \tag{40}$$

$$f_m(H) = \sum_{g \in G} \sigma_T^g {T^g}_m + \sigma_I I_m(H) + \sigma_S S_m \ , \tag{41}$$

for stability.

Predictive performance of models. For each model, we train parameters by maximizing data likelihood (Eq. 25), and compare the performance of the models in predicting the observed mutation frequencies in tumors (Supplementary Table 2) as well as non-tumor mutated cells for p53 (Supplementary Table 5). To compare between the models M of different complexity, which corresponds to the number of parameters, we utilize both the Bayesian Information Criterion (BIC) and the Aikake Information Criterion (AIC):

$$\text{AIC} = 2(k - \ln(\hat{L}(D_m|\Theta_M^*))), \quad \text{BIC} = k \ln(n) - 2 \ln(\hat{L}(D_m|\Theta_M^*) \ , \tag{42}$$

where k is the number of parameters, n is the number of data points being fit, and Θ_M^* is the set of parameters that maximizes the likelihood for model M (Eq. 25). BIC has a higher penalty for the number of parameters in a model for our case where there are many mutation frequency data points being fit. Each version of the fitness model is assigned an AIC and a BIC value, which depends on the number

of parameters, the number of datapoints being fit (for BIC), and how well the data is fit. Model selection can be further justified by calculating the relative likelihood of models with respect to a reference criterion value corresponding to a reference model. We justify model selection by calculating the relative likelihood of models with respect to the two-parameter reference model (Eq. 14) , which can be expressed as:

$$r_M = \exp[(C_{\mathsf{REF}} - C_M)/2] \, , \tag{43}$$

where r_M is the relative likelihood value corresponding to model M, C_M is the criterion value corresponding to model M, and C_{REF} is the criterion value corresponding to the reference model. The criterion value can either be from AIC or BIC. The relative likelihood value quantifies how likely model M minimizes information loss with respect to the reference model. We evaluate r_M for all alternative models with respect to our two-component minimal fitness model, (Eq. 14), which is used as the reference model throughout the manuscript.

We additionally compute the rank (Spearman) and linear (Pearson) correlation coefficients and p-values for the observed and predicted frequencies, x and $\hat{x}$, respectively. The p-values are computed assuming a null distribution of correlation values derived from two independent t-distributions using the exact Pearson and Spearman probability density functions in the Python *stats.pearsonr* and *stats.spearmanr* functions from the *scipy* package.

Datasets. In the models we developed across tumor suppressors and oncogenes, which did not have concentration information, we evaluated the performance of all possible models with parameters fit on TCGA haplotypes and separately on TCGA and COSMIC mutation distributions for each gene. We fit models on both TCGA and COSMIC mutation distributions since the mutation distributions for many genes were less consistent between the databases as compared to p53. When comparing COSMIC and TCGA, we filtered out the mutations from COSMIC that also appear in TCGA. When comparing TCGA and IARC, we filtered out the mutations from IARC that also appear in TCGA.

Predictive performance. For p53, the two component minimal fitness model (Eq. 14) leads to predicted mutation frequencies that are strongly correlated to the observed mutation distribution in tumors. Moreover, consistently, as evaluated across the measures and reported in Supplementary Table 2, models including both the functional and immune fitness components over-perform partial models, leading to predicted mutation frequencies that are strongly correlated to the observed mutation distribution. The addition of the immunogenicity component reduces the KL divergence of the predicted mutation frequencies with respect to the observed mutation frequencies and, despite increased model complexity, significantly improves model performance. Evaluation of the relative likelihood ratio (Eq. 43) demonstrates that the partial models have virtually no probability of minimizing information loss with respect to the minimal two-parameter model (Eq. 14). Moreover, while the two-parameter minimal model is highly predictive, we observe further increased predictive power of the extended models. These results illustrate how the proposed fitness model framework can be extended and can be used to gauge the importance of various phenotypes.

In predicting the non-neoplastic mutation distribution, addition of the immune component improved predictions to a lower degree than for the neoplastic mutation distribution. In the neoplastic setting, a combined model is $\simeq 10^7$ times more likely to be a more appropriate model, whereas in the non-neoplastic setting the benefit was zero for a comparable sample size (2,764 mutation occurrences in TCGA versus 3,451 mutation occurrences in non-neoplastic settings) as determined via BIC (Supplemen-

tary Table 5). This suggested that the role of the immune system in non-neoplastic cells may be smaller, which possibly depends on other genetic mutations, its environment, and how close the lesion is to becoming a neoplastic tumor.

For the models we tested across all examined cancer driver genes, we determined the appropriate model complexity via the Bayesian Information Criteria for both TCGA- and COSMIC-fit mutation frequency fitness models. We find that the appropriate model differs across the examined tumor suppressors and oncogenes, and that there is a positive relationship between the complexity of the model and the variance in the mutation frequencies for a particular gene. We find that genes with increased variance in mutation frequency are best explained by immunogenicity-only and combined models. These results illustrate the unique driving forces behind the mutation frequencies across diverse tumor suppressors and oncogenes, and show how minimal models successfully predict the mutation frequencies across these commonly mutated genes central in cancer development.

Effect of the number of simulated haplotypes. We train p53 models on germline TCGA HLA haplotypes and TCGA mutations. TCGA haplotypes are directly inferred from TCGA samples and are not simulated. To investigate the effect of the number of haplotypes on the modeling results, we applied the same model weights to models with populations in the half-open interval [1, 10,000) simulated haplotypes in 100 haplotype steps, and in each case quantified the Kullback-Leibler divergence.

Internal validation. In the fitness model, each p53 mutation is assigned an effective background mutation rate, functional phenotype, and immune phenotype, where the phenotypes are linked by mutant p53 concentration. We investigated the consequences of shuffling these components on the model fitting. We posited that the fitness model was only appropriate based on the available experimental and computational data for *TP53* mutations, and randomly shuffling these values should render phenotype data which the fitness model can not appropriately fit. For each internal validation step, we randomly permuted the background mutational frequencies, functional phenotypes, and immune phenotypes 1,000 times and attempted each time to fit the reference two-parameter model (Eq. 14). In each iteration, the minimized Kullback-Leibler divergence is always an order of magnitude larger than the results with non-shuffled data, and we found that in no case were we able to fit a model as well as with the non-permuted data. This suggests that the fitness model presented would not be appropriate for randomly-generated datasets.

Relative immune weight. To quantify the relative contribution of the fitness components, we refactor our fitness expression to a form that is equivalent for predicting mutation frequency. To do so, we standardize the T_m and I_m distributions across mutations and haplotypes to an equivalent relative fitness form:

$$\begin{aligned} \tilde{f}_m(\{\sigma_T, \sigma_I\}, H) &= \sigma_T s_T \left(\frac{T_m - \mu_T}{s_T}\right) + \sigma_I s_I \left(\frac{I_m - \mu_I}{s_I}\right) \\ &= \sigma_T s_T \left(\frac{T_m}{s_T}\right) + \sigma_I s_I \left(\frac{I_m}{s_I}\right) - \left[\frac{\sigma_T \mu_T}{s_T} + \frac{\sigma_T \mu_T}{s_T}\right] \\ &= (\sigma_T)' \left(\frac{T_m}{s_T}\right) + (\sigma_I)' \left(\frac{I_m}{s_I}\right) - \left[\frac{\sigma_T \mu_T}{s_T} + \frac{\sigma_I \mu_I}{s_I}\right] , \end{aligned} \tag{44}$$

where $\mu_{T,I}$ and $s_{T,I}$ are the means and standard deviations of the T_m and I_m distributions, respectively, across mutations and haplotypes, and $(\sigma_T)' = s_T\sigma_T$ and $(\sigma_I)' = s_I\sigma_I$. Note that the fitness is translationally invariant, so the final constant term is not relevant for predicting the mutant frequencies. Therefore, the fitness as expressed in Eq. 44 is equivalent to the original fitness expression (Eq. 14) for predicting mutation frequencies, as the only difference between them is a constant.

The sum of the two rescaled fitness weights correspond to a particular amplitude A'. Note that both fitness expressions from Eq. 14 and Eq. 44 have only one degree of freedom despite the fact that there are two parameters, since $(\sigma_T)' = A' - (\sigma_I)'$. Therefore, Eq. 44 can be written as a linear function of $(\sigma_I)'$ only. Knowing this, we can define the relative immune weight ν_I as:

$$\nu_I = \frac{|(\sigma_I)'|}{|(\sigma_T^*)'| + |(\sigma_I^*)'|}\,, \tag{45}$$

where $(\sigma_T^*)'$ and $(\sigma_I^*)'$ are optimized standardized weights. We derive $s_T = 0.178$ and $s_I = 0.0106$ from the full TCGA mutant *TP53* T_m and $I_m(H)$ distributions, respectively, across mutations, haplotypes, and tissues, as this is the data with which we train our fitness model.

To determine the optimal model for a cohort, we vary the parameter ν_I over the interval $[0, 1]$ to determine the relative importance of the immune component to an optimized model. We do so by recomputing the logrank scores for Kaplan-Meier curves separated on the median mutant p53 total fitness defined for each ν_I value.

Modeling trade-offs for *KRAS*

Utilizing the detailed functional information available for a number of *KRAS* hotspot mutations[25], we inferred the oncogenicity and the immunogenicity of these *KRAS* hotspot mutations in TCGA PAAD samples. Importantly, the only functional component which was predictive for fitting the *KRAS* mutational distribution was the downstream RAF protein effector binding. Therefore, for the functional "oncogenic" component, we determined the probability of a particular mutant KRAS binding downstream RAF effector protein in the MAPK pathway in a non-cooperative fashion, normalized by the number of *KRAS* alleles and assuming equal number of wild-type and mutant *KRAS* alleles as well as fully-active mutant KRAS. The "functional probability" component summarizes the likelihood of active, mutant KRAS binding RAF protein and transducing cell growth signaling:

$$P_{\text{RAF}} = \frac{L_{\text{KRAS}}}{L_{\text{KRAS}} + K_{\text{RAF}}}\,, \tag{46}$$

where L_{KRAS} is the inferred concentration of mutant KRAS in a particular cancer cell, and K_{RAF} is the provided dissociation constant for KRAS-RAF protein binding from Ref. 25. For the immune component, we inferred the effective probability of mutant KRAS nonamer peptides being presented on matched HLA-I molecules, in a manner similar to Eq. 16. The "avoidance of neoantigen presentation" component is therefore defined as $1 - I_m(H)$, where I_m refers to Eq. 16 and H is a germline MHC-I haplotype.

There is no RPPA proteomic data available for *KRAS* in TCGA. In order to address this, we inferred the concentrations of KRAS in TCGA PAAD samples using *KRAS* RNA expression, calibrated using known wild-type KRAS concentrations in a WT/WT KRAS SW48 cell line. We infer the wild-type KRAS concentrations from Ref. 83, using the parental wild-type cell line. We assume a cell diameter of 20 micrometers[78], a typical *KRAS* ploidy of two which means suggest $\simeq 10^5$ KRAS protein molecules per allele, and a spherical cell shape. In brief, we assumed that all RNA expression was strongly linearly correlated to protein expression. Next, since the SW48 cell line is derived from a colon cancer, we calibrated the RNA expression to an expected concentration value across wild-type KRAS TCGA COAD tumors. This was done in an analogous way as for p53, where we inferred concentration using wild-type p53 BRCA RPPA data calibrated using a breast cancer-derived cell line with known wild-type p53 concentration. From this, we obtain an expected concentration of KRAS for each TCGA PAAD tumor cell. We further normalize by the number of alleles, assuming equal numbers of wild-type and mutant *KRAS* alleles.

As the protein concentration goes into both the oncogenic and immunogenic terms, cancer cells which upregulate mutant KRAS, for the purpose of increased cell growth, do so at the cost of increasing the concentration of the mutant antigen, implying a trade-off between the oncogenic potential of a mutant and its immune selection in upregulated oncogenes.

Validating trade-offs with ATAC-seq and RNA

We predict functional fitness based on the yeast functional assay (see Section **Inference of apparent dimer dissociation constants**). We estimate downstream functional capacities of mutant p53 on target gene RNA expression in a tumor using ATAC-seq (Assay for Transposase-Accessible Chromatin with high-throughput sequencing) and RNA-seq data in matched TCGA samples. Previous work performed ATAC-seq on 423 TCGA samples across 23 cancer types, predominantly breast cancer[79]. We leverage ATAC-seq transcription factor footprinting, using three *TP53* motifs (M3698_1.02, M1929_1.02, and M3699_1.02) for which transcription depth and flank are measured. Increased depth indicates higher transcription factor occupancy and increased flank indicates increased increased chromatic accessibility by other factors[80].

The flanking accessibility (A_F) and footprint depth (D_F) are computed for each *TP53* motif (M) as follows:

$$A_F^M = \log_2 \left[\frac{\text{Flank Height}}{\text{Background}} \right] , \tag{47}$$

and

$$D_F^M = \log_2 \left[\frac{\text{Footprint Base}}{\text{Flank Height}} \right] . \tag{48}$$

We compute a lack of DNA binding score (N_F^M) for p53 for each motif M as:

$$N_F^M = 2^{\frac{D_F^M}{A_F^M}} . \tag{49}$$

As this value increases, either the depth increases and/or the flank decreases, indicating a lack of binding compared to background. For each sample with available data, we identified samples with one mutant *TP53* allele. We extract the depth, flank, and determine the combined lack of binding score N_F^M for each motif, which we use as a proxy for the likelihood p53 is not binding its DNA target motif. For each mutation, we define the effective lack of DNA binding score as the harmonic mean of the lack of binding scores across the three motifs in order to control for large outlier values:

$$N_F = \frac{3}{\sum_M 2^{-\frac{D_F^M}{A_F^M}}} . \tag{50}$$

We consider RNA expression in Transcripts per Million (TPM) of the eight p53 target genes previously examined in the yeast assay (*WAF1*, *MDM2*, *BAX*, *h1433s*, *AIP1*, *GADD45*, *NOXA*, and *P53R2*). There are 373 TCGA samples with matched ATAC-seq and RNA-seq data.

Independently, we also consider the chromatin accessibility on regulatory regions for the eight target genes where such data is available[79]. This was the case for only six target genes (*WAF1*, *BAX*, *h1433s*, *AIP1*, *GADD45*, and *NOXA*). Each gene may have multiple regulation sites, and each site has an associated number of Tn5 transposase insertion events which correlate to the site's chromatin accessibility. We transform the accessibility of these regulation sites into a probability distribution as follows. First, we define the chromatin accessibility of each gene G as G_A, which is the sum of the insertions across all regulatory sites:

$$G_A = \sum_r I_r \ , \tag{51}$$

where r is a regulatory site and I_r is the number of Tn5 transposase insertions corresponding to a regulatory site. This takes into account both the number of regulatory sites and the accessibility of these sites. For each sample, we define the median target gene accessibility S_A as the median number of gene insertions across all of the target genes' regulatory sites:

$$S_A = \text{median}_G \left(G_A \right) \ . \tag{52}$$

Finally, we transform the distribution of S_A into a probability distribution via the softmax function, where $P(A)$ is the probability the p53 target genes are accessible in a particular sample:

$$P(A) = \frac{\exp S_A}{\sum_S \exp S_A} \ . \tag{53}$$

We then define the probability of p53 binding target DNA $P(B)$ as follows:

$$P(B) = \frac{P(B|A)P(A)}{P(A|B)} \ , \tag{54}$$

where $P(B \mid A)$ is the probability of p53 binding DNA given it is accessible, which is derived from the yeast assay and the mutant's typical concentration (see Sections **Inference of apparent dimer dissociation constants**, **Inference of tissue- and mutant-specific concentrations**, and **Fitness Model**), $P(A)$ is the sample's target gene regulatory site accessibility probability, and $P(A \mid B) = 1$, where if p53 is binding DNA then it follows that the DNA is by accessible. Therefore, the probability of p53 binding DNA is conditioned on the probability of the target genes having sufficient chromatin accessibility.

Patient Data

Immunotherapy-treated non-small cell lung cancer cohort. Patients were those with metastatic non-small cell lung cancer (NSCLC) treated with PD-(L)1 blockade-based immunotherapy between 2013-2019. Those treated with concurrent PD-(L)1 + cytotoxic chemotherapy were excluded. To be included, patients had to have molecular next-generation sequencing results by MSK-IMPACT as well as available outcomes data from their response to PD(L)1 therapy. Objective overall response and progression-free survival outcomes were determined by RECIST, performed by a blinded thoracic radiologist. Patients who did not progress were censored at the time of their last available imaging assessment. Overall survival was determined from the start of PD-(L)1 blockade until date of death; those who were still alive were censored at the time of last contact.

National Cancer Institute Li-Fraumeni Syndrome cohort. A total of 82 individuals carrying either pathogenic or likely pathogenic missense germline *TP53* variants from the National Cancer Institute (NCI) LFS cohort (NCT01443468; http://lfs.cancer.gov)[81] were included. All participants or their legal guardians signed informed consent. As of March 24, 2020, 52 carriers had developed at least one cancer while 30 had remained cancer-free. Non-melanoma skin cancers and HPV-associated high grade dysplasias were excluded from the cancer count. Genotyping was conducted using the Illumina Infinium Global Screening Array-24 (Illumina Inc. San Diego) at the Cancer Genomics Research Laboratory (CGR) in the Division of Cancer Epidemiology and Genetics (DCEG). HLA alleles were imputed with the tool HIBAG[82] using a model trained for European ancestry.

Experimental Methods

Peptide predictions. The HLA molecules predicted to present the R175H and R248Q/W hotspot peptides using NetMHC 3.4[50–52] are reported in Supplementary Table 6. The HLA-A*02:01 allele is the most common HLA-I in TCGA. We aimed to infer differential potential immunogenicity between *TP53* R175H and R248Q/W mutations, as these hotspots lie on different end of the trade-off between loss of function and potential neoantigen immunogenicity. We inferred all mutant peptides of 8-14 amino acids in length that cover the R175H and R248Q/W mutations and predicted IC50 affinities to HLA-A*02:01 using the NetMHC 3.4. All peptides with predicted affinities less than 500 nM are presented in Supplementary Table 6. Only peptides corresponding to the R248Q/W mutations passed this filter, and we used the 10-mer peptides for the in vitro assays, as they were more likely to be presented. For R175H, we considered the HMTEVVRHC peptide, as previous work implied this peptide can be presented on HLA-A*02:01[33] although the predicted affinity was 10716 nM.

T2 binding assay. The TAP2-deficient human lymphoblastoid cell line T2 was maintained in RPMI-1640 supplemented with 7.5% FBS, NEAA, 2 mM L-glutamine and penicillin/streptomycin. Prior to assay setup, T2 cells were washed three times in serum-free RPMI-1640 and then plated at a concentration of 1x106/mL in serum-free RPMI-1640 with 5 μg/mL recombinant human (rh)β2 microglobulin (Sigma-Aldrich, cat. no. 475828) and 1, 10 or 100 μg/mL of peptide (>85% purity, Genscript) or DMSO as vehicle control and incubated overnight. The following day, cells were washed and stained with a fixable viability dye (Zombie NIR, 1:8000, BioLegend, cat. no. 423106) in PBS for 15 min on ice. Cells were then washed and stained with a FITC-conjugated anti-human HLA-A*02 antibody (clone BB7.2, 1:100, BD Biosciences, cat. no. 551285) for 30 min on ice in PBS. After staining, cells were washed and resuspended in PBS for acquisition on a 4-laser Aurora full spectrum cytometer (UV-V-B-R, Cytek). Data were analyzed using FlowJo software (version 10.7.1).

Human samples. All patients and healthy donors signed an approved informed consent before providing tissue samples. Patient samples were collected on a tissue-collection protocol approved by the MSKCC Institutional Review Board. Peripheral blood mononuclear cells (PBMCs) from HLA-A*02:01 healthy donors and patients with *TP53* R175H or R248Q mutant bladder or ovarian cancer were isolated from whole blood collected in CPT tubes containing sodium heparin (BD Vacutainer) according to the manufacturer's instructions. PBMCs from cancer patients were cryopreserved in FBS containing 10% DMSO until use. PBMCs from healthy donors were plated in 10 cm tissue culture dishes at 4-6x106 cells/mL in RPMI-1640, supplemented with 1% human serum (pooled male AB, Sigma-Aldrich, cat. no. H4522), 10 mM HEPES, 2 mM L-glutamine, and 50 μM 2-β-mercaptoethanol and incubated at 37C for one hour. Non-adherent cells were washed off with PBS and cryopreserved in FBS containing 10% DMSO until further use. Adherent cells were cultured for 7 days in RPMI-1640 with 1% human serum, 1000 IU/mL rhGM-CSF, and 500 IU/mL rhIL-4 to induce differentiation of monocytes into monocyte-derived dendritic cells (mDCs). CD4+ and CD8+ T-cells were isolated from the non-adherent cell fraction using human CD8 Microbeads (Miltenyi, cat. no. 130-045-201) and the human CD4+ T-cell Isolation Kit (Miltenyi, cat. no. 130-096-533) according to the manufacturer's instructions. CD4+ T-cells were activated with 10 μg/mL PHA and cultured in the presence of 10 IU/mL rhIL-2 and 20 ng/mL rhIL-7 for one week before using them as CD4+ Th-APCs in peptide restimulation assays.

***In vitro* peptide stimulation assays.** We used two types of in vitro peptide stimulation assays: one for inducing *de novo* priming of mutant p53 specific T-cell responses from healthy donors and one for

recalling memory responses against mutant p53 peptides in T-cells from patients bearing mutant p53 tumor lesions. To induce functional *de novo* priming of human CD8+ T-cells, we developed an optimized *in vitro* restimulation system (method manuscript in preparation). Briefly, CD8+ T-cells from HLA-A*02:01 healthy donors were stimulated with autologous mDCs pulsed with 10 µg/mL p53 peptides (>85% purity, Genscript), CEF (CEF-Class I peptide pool, 1:20, CTL), 1 µg/mL 15-mer HIV GAG peptide pool (JPT), or DMSO at a 5:1 ratio in RPMI-1640 supplemented with 10% FBS, NEAA, 2 mM L-glutamine, penicillin/streptomycin, 1 mM sodium pyruvate, and 50 µM β-mercaptoethanol (complete media) in the presence of 100 IU/mL rhIL-2 and 10 ng/mL rhIL-15. After one week of culture, cells were washed, and re-stimulated with peptide-pulsed, PHA-activated autologous CD4+ Th-APCs at a 1:1 ratio. Cultures were maintained in 100 IU/mL rhIL-2 and 10 ng/mL rhIL-15 for a second week. Cells were then washed and incubated with the specific peptides before intracellular cytokine staining by flow cytometry. To recall mutant p53 T-cell responses, patients' PBMCs were stimulated with 10 µg/mL R175H and/or R248Q p53 (>85% purity, Genscript), CEF (CEF-Class I peptide pool, 1:20, CTL) as positive control, or DMSO as negative vehicle control in complete media in the presence of 10 IU/mL rhIL-2 and 10 ng/mL rhIL-15. Cells were restimulated with the respective peptides on day 7, and cultures were maintained with rhIL-2 and rhIL-15 for a second week. On day 15, cells were washed, restimulated with the specific peptides before intracellular cytokine staining by flow cytometry.

Intracellular cytokine staining by flow cytometry. Monensin (1-2 µM, BD GolgiStop, BD Pharmingen) was added 1 hour after the last peptide restimulation to inhibit intracellular protein transport and cultures were incubated for additional 5 hours. Cells were then washed and stained with an eFluor 506 (1:1000, eBioscience, cat. no. 65-0866-18) or Zombie NIR (1:8000, BioLegend, cat. no. 423106) fixable viability dye in PBS for 15 minutes on ice, followed by a 15-minute incubation with human Fc blocking reagent (1:10, Miltenyi) in 2% FBS PBS on ice, before staining with the following fluorochrome-conjugated surface antibodies: anti-human CD3-BUV395 (1:100, BD Biosciences, cat. no. 740283), anti-human CD4-BV650 (1:50, BD Biosciences, cat. no. 563875) or CD4-AlexaFluor700 (1:50, Invitrogen, cat. no. 56-0047-42), and anti-human CD8-BUV563 (1:50, BD Biosciences, cat. no. 612914) or CD8-AlexaFluor647 (1:50, BD Biosciences, cat. no. 557708), anti-human CD45RA-BUV737 (1:100, BD Biosciences, cat. no. 564442), and anti-human CD62L-PE (1:100, BD Biosciences, cat. no. 555544). After 40-minute incubation on ice, cells were washed and subsequently fixed and permeabilized using the FoxP3/Transcription Factor Staining Buffer Set (Thermo Fisher Scientific, cat. no. 00-5523-00). Intracellular staining was performed in permeabilization buffer for 45 minutes on ice with the following antibodies: anti-human IFN-γ-FITC (1:50, Invitrogen, cat. no. BMS107FI), anti-human TNF-α-PE-Cy7 (1:50, BD Biosciences, cat. no. 557647) and anti-human Ki67-APC-eFluor 780 (1:1600, Invitrogen, cat. no. 47-5698-82). Cells were washed in permeabilization buffer and resuspended in PBS for acquisition on a 4 laser Aurora full spectrum cytometer (UV-V-B-R, Cytek). Data were analyzed using FlowJo software (version 10.7.1).

Multiplex Identification of Antigen-Specific T-Cell Receptors (MIRA) assay. To compare the relative immune fitness of *TP53* mutations depending on the position of their amino acid substitutions, we used MIRA to search for TCRs against mutant p53 in naive CD8 T-cell repertoires of healthy donors. MIRA combines conventional immunological techniques with high-throughput TCR sequencing to identify antigen specific T-cells in high-throughput through the sorting and sequencing of T-cells activated in response to pools of peptide epitopes[83].

We synthesized 40 distinct 9-11 length peptide epitopes that encompassed common p53 mutations at positions pR175 (H), pR248 (Q), pR273 (C/H/L), and pR282 (W) and which were predicted to bind to at

least one of 60 common HLA class I alleles by NetMHCpan version 4.1[84] (Supplementary Table 7). Peptide synthesis was performed by GenScript (Piscataway, NJ). The 40 peptides were pooled in a combinatorial fashion as described previously[83], where peptides with high sequence similarity were grouped together into discrete antigen sets. Each antigen set was placed in a unique subset of 6 out of 11 peptide pools labelled A-K, hereafter referred to as the antigen's occupancy.

We acquired Leukopaks from 107 healthy donors from a variety of commercial sources (AllCells, Alameda CA & Bloodworks Northwest, Seattle WA). Donors represented diverse HLA Class I backgrounds, encompassing 25 distinct HLA-A alleles, 46 HLA-B alleles, and 20 HLA-C alleles at 4 digit typing (Supplementary Table 7). 100/107 donors had at least one A*02:01 allele. There were 103 unique MHC-I haplotypes. We conducted a total of 222 MIRA experiments; on average 2 experiments per donor.

MIRA experiments were performed as follows: naive CD8 (nCD8) T-cells were isolated from donor Leukopaks and 30-200 million nCD8s were co-cultured for 12-14 days with monocyte-derived dendritic cells pulsed with the entire set of query peptides in the presence of cytokines GM-CSF/IL-4/IFN-g and LPS. T-cells were supplemented with IL-7 and IL-15 on day 3 of the expansion. Following a 12-14 day expansion, the T-cell culture was split into replicate aliquots and T-cells were re-stimulated with MIRA-formatted peptide pools at 37C for 16 hours. Sorting was done on CD3+CD8+CD137+ T-cells and followed similar preparation and sequencing of the TCRb locus as previously reported[85]. T-cell presence was assessed by aggregating the behaviour of specific TCRb sequences across sorted pools and we utilized a non-parametric Bayesian model described previously[85] to identify T-cell clonotypes with read count patterns consistent with enrichment in 6 of the 11 replicate antigen exposures[83].

We considered all TCR-antigen associations with a posterior probability of >= 0.5 to represent a significant response to the antigen at that occupancy, then counted the number of TCRs that responded to antigens with each of the p53 p175, p248, p273, and p282 mutations. To permit fair comparison of the number of TCRs yielded between each of the *TP53* positional mutants, we calculated each donor's average count of TCRs yielded per antigen peptide by (1) the number of peptides in the MIRA antigen set (i.e. the number of putative epitopes at that occupancy), (2) the number of MIRA antigens (i.e. occupancies) representing each of the four *TP53* positional mutants, and (3) each donor's number of experiments. This procedure yielded a single value representing each of the 107 donors' average number of TCRs yielded per antigen peptide, for each of the *TP53* p175, p248, p273, and p282 MIRA antigen groups.

We reasoned that *TP53* positional mutation antigen groups with lower immune fitness should yield higher normalized TCR yield from these 107 healthy donors. To test for significant differences in normalized TCR yield, we conducted a two-sided Mann-Whitney U Tests on normalized TCR yield values for each pairwise combination of p175, p248, p273, and p282.

References

39. Lunter, G. & Hein, J. A nucleotide substitution model with nearest-neighbour interactions. *Bioinformatics* **20**, i216–i223 (2004). URL `https://doi.org/10.1093/bioinformatics/bth901`.

40. Wheeler, D. L. *et al.* Database resources of the national center for biotechnology information. *Nucleic Acids Research* **36**, D13–D21 (2007).

41. Cingolani, P. *et al.* A program for annotating and predicting the effects of single nucleotide polymorphisms, SnpEff: SNPs in the genome of Drosophila melanogaster strain w^{1118}; iso-2; iso-3. *Fly* **6**, 80–92 (2012).

42. Ashkenazy, H. *et al.* ConSurf 2016: an improved methodology to estimate and visualize evolutionary conservation in macromolecules. *Nucleic Acids Research* **44**, W344–W350 (2016).

43. Berezin, C. *et al.* ConSeq: the identification of functionally and structurally important residues in protein sequences. *Bioinformatics* **20**, 1322–1324 (2004).

44. Consortium, T. U. UniProt: The universal protein knowledgebase in 2021. *Nucleic Acids Research* **49**, D480–D489 (2021).

45. Suzek, B. E. *et al.* UniRef clusters: a comprehensive and scalable alternative for improving sequence similarity searches. *Bioinformatics* **31**, 926–932 (2015).

46. Li, W. & Godzik, A. Cd-hit: a fast program for clustering and comparing large sets of protein or nucleotide sequences. *Bioinformatics* **22**, 1658–1659 (2006).

47. Fu, L., Niu, B., Zhu, Z., Wu, S. & Li, W. CD-HIT: accelerated for clustering the next-generation sequencing data. *Bioinformatics* **28**, 3150–3152 (2012).

48. Katoh, K. & Standley, D. M. MAFFT multiple sequence alignment software version 7: improvements in performance and usability. *Molecular Biology and Evolution* **30**, 772–780 (2013).

49. Pupko, T., Bell, R. E., Mayrose, I., Glaser, F. & Ben-Tal, N. Rate4Site: an algorithmic tool for the identification of functional regions in proteins by surface mapping of evolutionary determinants within their homologues. *Bioinformatics* **18**, S71–S77 (2002).

50. Nielsen, M. *et al.* Reliable prediction of T-cell epitopes using neural networks with novel sequence representations. *Protein Science* **12**, 1007–1017 (2003).

51. Lundegaard, C., Lund, O. & Nielsen, M. Accurate approximation method for prediction of class I MHC affinities for peptides of length 8, 10 and 11 using prediction tools trained on 9mers. *Bioinformatics* **24**, 1397–1398 (2008).

52. Lundegaard, C. *et al.* NetMHC-3.0: accurate web accessible predictions of human, mouse and monkey MHC class I affinities for peptides of length 8–11. *Nucleic Acids Research* **36**, W509–W512 (2008).

53. Andreatta, M. & Nielsen, M. Gapped sequence alignment using artificial neural networks: application to the MHC class I system. *Bioinformatics* **32**, 511–517 (2016).

54. Weinberg, R. L., Veprintsev, D. B. & Fersht, A. R. Cooperative binding of tetrameric p53 to DNA. *Journal of Molecular Biology* **341**, 1145–1159 (2004).

55. Weinberg, R. L., Veprintsev, D. B., Bycroft, M. & Fersht, A. R. Comparative binding of p53 to its promoter and DNA recognition elements. *Journal of Molecular Biology* **348**, 589–596 (2005).

56. Weinberg, R. L., Freund, S. M., Veprintsev, D. B., Bycroft, M. & Fersht, A. R. Regulation of DNA binding of p53 by its C-terminal domain. *Journal of Molecular Biology* **342**, 801–811 (2004).

57. He, F. *et al.* Interaction between p53 N terminus and core domain regulates specific and nonspecific DNA binding. *Proceedings of the National Academy of Sciences* **116**, 8859–8868 (2019).

58. Cain, C., Miller, S., Ahn, J. & Prives, C. The N terminus of p53 regulates its dissociation from DNA. *Journal of Biological Chemistry* **275**, 39944–39953 (2000).

59. Friedler, A., Veprintsev, D. B., Freund, S. M., Karoly, I. & Fersht, A. R. Modulation of binding of DNA to the C-terminal domain of p53 by acetylation. *Structure* **13**, 629–636 (2005).

60. Jordan, J. J. *et al.* Low-level p53 expression changes transactivation rules and reveals superactivating sequences. *Proceedings of the National Academy of Sciences* **109**, 14387–14392 (2012).

61. Bouaoun, L. *et al.* *TP53* variations in human cancers: new lessons from the IARC *TP53* database and genomics data. *Human Mutation* **37**, 865–876 (2016).

62. Li, J. *et al.* Explore, visualize, and analyze functional cancer proteomic data using the cancer proteome atlas. *Cancer Research* **77**, e51–e54 (2017).

63. Li, J. *et al.* TCPA: a resource for cancer functional proteomics data. *Nature Methods* **10**, 1046–1047 (2013).

64. Raine, K. M. *et al.* ascatNgs: Identifying somatically acquired copy-number alterations from whole-genome sequencing data. *Current Protocols in Bioinformatics* **56**, 15–9 (2016).

65. Tate, J. G. *et al.* COSMIC: the catalogue of somatic mutations in cancer. *Nucleic Acids Research* **47**, D941–D947 (2019).

66. Grossman, R. L. *et al.* Toward a shared vision for cancer genomic data. *New England Journal of Medicine* **375**, 1109–1112 (2016).

67. Landau, D. A., Carter, S. L., Getz, G. & Wu, C. J. Clonal evolution in hematological malignancies and therapeutic implications. *Leukemia* **28**, 34–43 (2014).

68. Dentro, S. C., Wedge, D. C. & Van Loo, P. Principles of reconstructing the subclonal architecture of cancers. *Cold Spring Harbor Perspectives in Medicine* **7**, a026625 (2017).

69. Khiabanian, H. *et al.* Inference of germline mutational status and evaluation of loss of heterozygosity in high-depth, tumor-only sequencing data. *JCO Precision Oncology* **2**, 1–15 (2018).

70. McGranahan, N. *et al.* Clonal status of actionable driver events and the timing of mutational processes in cancer evolution. *Science Translational Medicine* **7**, 283ra54–283ra54 (2015).

71. Chan, W. M., Siu, W. Y., Lau, A. & Poon, R. Y. How many mutant p53 molecules are needed to inactivate a tetramer? *Molecular and Cellular Biology* **24**, 3536–3551 (2004).

72. Thorsson, V. *et al.* The immune landscape of cancer. *Immunity* **48**, 812–830 (2018).

73. Gonzalez-Galarza, F. F. *et al.* Allele frequency net database (AFND) 2020 update: gold-standard data classification, open access genotype data and new query tools. *Nucleic Acids Research* **48**, D783–D788 (2020).

74. Gragert, L., Madbouly, A., Freeman, J. & Maiers, M. Six-locus high resolution HLA haplotype frequencies derived from mixed-resolution DNA typing for the entire US donor registry. *Human Immunology* **74**, 1313–1320 (2013).

75. Bullock, A. N., Henckel, J. & Fersht, A. R. Quantitative analysis of residual folding and DNA binding in mutant p53 core domain: definition of mutant states for rescue in cancer therapy. *Oncogene* **19**, 1245–1256 (2000).

76. Tan, Y. & Luo, R. Structural and functional implications of p53 missense cancer mutations. *PMC Biophysics* **2**, 5 (2009).

77. Chen, Y., Dey, R. & Chen, L. Crystal structure of the p53 core domain bound to a full consensus site as a self-assembled tetramer. *Structure* **18**, 246–256 (2010).

78. Mageean, C. J., Griffiths, J. R., Smith, D. L., Clague, M. J. & Prior, I. A. Absolute quantification of endogenous Ras isoform abundance. *PLoS One* **10**, e0142674 (2015).

79. Corces, M. R. *et al.* The chromatin accessibility landscape of primary human cancers. *Science* **362** (2018).

80. Baek, S., Goldstein, I. & Hager, G. L. Bivariate genomic footprinting detects changes in transcription factor activity. *Cell Reports* **19**, 1710–1722 (2017).

81. Mai, P. L. *et al.* Risks of first and subsequent cancers among *TP53* mutation carriers in the National Cancer Institute Li-Fraumeni syndrome cohort. *Cancer* **122**, 3673–3681 (2016).

82. Zheng, X. *et al.* HIBAG—HLA genotype imputation with attribute bagging. *The Pharmacogenomics Journal* **14**, 192–200 (2014).

83. Klinger, M. *et al.* Multiplex identification of antigen-specific T cell receptors using a combination of immune assays and immune receptor sequencing. *PLoS One* **10**, e0141561 (2015).

84. Jurtz, V. *et al.* NetMHCpan-4.0: improved peptide–MHC class I interaction predictions integrating eluted ligand and peptide binding affinity data. *The Journal of Immunology* **199**, 3360–3368 (2017).

85. Snyder, T. M. *et al.* Magnitude and dynamics of the T-cell response to SARS-CoV-2 infection at both individual and population levels. *MedRxiv* (2020).
